# Benthic exometabolites and their ecological significance on threatened Caribbean coral reefs

**DOI:** 10.1038/s43705-022-00184-7

**Published:** 2022-10-17

**Authors:** Laura Weber, Melissa Kido Soule, Krista Longnecker, Cynthia C. Becker, Naomi Huntley, Elizabeth B. Kujawinski, Amy Apprill

**Affiliations:** 1 Marine Chemistry and Geochemistry Department, Woods Hole Oceanographic Institution, Woods Hole, MA 02543 USA; 2 MIT-WHOI Joint Program in Oceanography/Applied Ocean Science and Engineering, Cambridge and Woods Hole, MA USA; 3 Marine and Environmental Science Department, University of the Virgin Islands, Charlotte Amalie West, St Thomas, Charlotte Amalie, VI 00802 USA; 4 Department of Biology, Pennsylvania State University, University Park, PA USA

**Keywords:** Water microbiology, Microbial ecology, Biogeochemistry

## Abstract

Benthic organisms are the architectural framework supporting coral reef ecosystems, but their community composition has recently shifted on many reefs. Little is known about the metabolites released from these benthic organisms and how compositional shifts may influence other reef life, including prolific microorganisms. To investigate the metabolite composition of benthic exudates and their ecological significance for reef microbial communities, we harvested exudates from six species of Caribbean benthic organisms including stony corals, octocorals, and an invasive encrusting alga, and subjected these exudates to untargeted and targeted metabolomics approaches using liquid chromatography-mass spectrometry. Incubations with reef seawater microorganisms were conducted to monitor changes in microbial abundances and community composition using 16 S rRNA gene sequencing in relation to exudate source and three specific metabolites. Exudates were enriched in amino acids, nucleosides, vitamins, and indole-based metabolites, showing that benthic organisms contribute labile organic matter to reefs. Furthermore, exudate compositions were species-specific, and riboflavin and pantothenic acid emerged as significant coral-produced metabolites, while caffeine emerged as a significant invasive algal-produced metabolite. Microbial abundances and individual microbial taxa responded differently to exudates from stony corals and octocorals, demonstrating that exudate mixtures released from different coral species select for specific bacteria. In contrast, microbial communities did not respond to individual additions of riboflavin, pantothenic acid, or caffeine. This work indicates that recent shifts in benthic organisms alter exudate composition and likely impact microbial communities on coral reefs.

## Introduction

Tropical coral reefs are highly productive ecosystems that, despite existing in generally oligotrophic waters, support an immense variety of life. Benthic organisms, like stony corals, octocorals, sponges, turf algae, and macroalgae, are holobiont organisms [1], hosting diverse microorganisms including bacteria and archaea, which in turn support the metabolism, growth, and health [2–5] of their eukaryotic hosts and drive the high productivity and rapid organic matter cycling on coral reefs [6–8]. Importantly, benthic algae and the symbiotic algae that live within coral tissue [9] fix carbon through photosynthesis and release a portion of these exudates directly into the surrounding seawater [10] or to the coral host [11, 12]. In corals, the translocated photosynthate from Symbiodinaceae fuels coral metabolism and excess carbon is excreted as dissolved or particulate (mucus) organic matter [13]. Sponge holobionts release metabolites, including amino acids and nucleosides, into the seawater through their exhalant [14] and these and other organic molecules are recycled back into the food web [15]. The combined and simultaneous release, consumption, and production of metabolites from benthic organisms results in a complex extracellular metabolite pool on reefs [14, 16].

Planktonic and particle-bound microorganisms overlying reefs play critical functions in community metabolism. Heterotrophic bacteria and archaea recycle dissolved and particulate organic matter released from benthic organisms, liberate limiting nutrients like nitrogen and phosphorous, and shunt carbon back into the food web [17, 18]. Photosynthetic picocyanobacteria tend to be abundant in reef seawater [19–21] and contribute to total reef productivity (reviewed within [6]). This benthic-pelagic coupling, likely driven by the exchange of benthic-derived organic matter, influences the planktonic microbial community overlying the reef. Large-scale changes in benthic reef composition covary with changes in the membership and abundance of seawater microorganisms. For example, macroalgal-covered reefs select for more copiotrophic, potentially pathogenic, heterotrophic bacteria than coral-dominated reefs [22, 23]. At the colony scale, corals influence the composition and function of surrounding seawater microbial communities [24, 25]. These feedbacks between the reef benthos and the overlying reef seawater microbial community may change as the composition of benthic organisms on reefs shifts in favor of more algae and octocorals over stony corals [26–28] in response to anthropogenic stressors like eutrophication, overfishing, and global climate change. The hypothesis presented here and mentioned in other work is that the quantity, quality, and composition of dissolved and particulate organic matter on reefs is linked to benthic community composition because benthic organisms release dissolved and particulate organic matter. Changes in the composition of available metabolites in reef seawater select for specific microorganisms, leading to alterations in the structure and function of reef microbial communities. Essentially, the dynamics of benthic-pelagic coupling on threatened reefs are modified because the primary sources of organic matter are changing.

In light of the importance of benthic exudates for reef functioning, efforts have been made to investigate their composition and influence on the reef microbial community. Benthic organisms, including macroalgae, turf algae, crustose coralline algae, and corals, release labile dissolved organic carbon (DOC), the rate of DOC release varies by species [29], and exudates differentially influence microbial communities [30–32]. In fact, dissolved exudates released from different coral and algae species are distinct from each other and enriched in differing organic macronutrients [33]. Additionally, corals release dissolved free amino acids [25, 34, 35], vitamins like riboflavin and other pterins [36], and compounds putatively identified as diterpenes, bacterial quorum sensing molecules, antibacterial compounds, and prenolipids [25]. These efforts support the hypothesis that benthic organisms release a variety of metabolites in their exudates but provide limited details about the variability and concentrations of individual metabolites released from different benthic organisms.

The differential release of metabolites from benthic reef organisms has particular significance for the Caribbean, where reefs have undergone a major phase shift from stony coral to octocoral dominance [28, 37, 38]. Octocorals exhibit different growth patterns compared to most stony corals; most have branching colony morphologies, and in some environments they grow faster than stony corals [39]. Octocoral recruits are also more abundant than stony corals on many Caribbean reefs due to their successful reproductive and settlement-related characteristics [40, 41]. Although the exact mechanisms are unclear, octocorals appear to be more resistant and resilient than stony corals to seawater temperature increases and may become the dominant anthozoan subclass on future Caribbean reefs [26]. Caribbean reefs are also experiencing enhanced benthic coverage of macro and crustose algae, including recent invasions of peyssonnelid algal crusts, such as the red alga *Ramicrusta textilis* [42–44]. These algal crusts overgrow corals and settle on available substrates, preventing the accumulation of native crustose coralline algae and their associated microbial biofilms that are necessary for the settlement and metamorphosis of stony coral larvae [45, 46]. Impacts of these shifts in benthic composition on the reef metabolite pool and subsequent microbial growth have yet to be investigated.

In this work, we sought to enhance our understanding of benthic-pelagic coupling on coral reefs by characterizing the exudates (i.e., the dissolved metabolites) released from important Caribbean benthic organisms and monitoring responses of microorganisms to these exudates. We applied targeted and untargeted metabolomics methods [47, 48] to exudates released from stony corals (*Porites astreoides*, *Siderastrea siderea*, *Pseudodiploria strigosa*), octocorals (*Plexauara homomalla*, *Gorgonia ventalina*), and the encrusting alga *Ramicrusta textilis*. Using these methods, we quantified known extracellular metabolites as well as compared metabolite features across species. We also conducted incubation experiments to examine microbial responses to complex mixtures of metabolites released from *P. astreoides* (stony coral) and *G. ventalina* (octocoral), as well as to individual metabolites displaying either widespread or organism-specific patterns (caffeine, pantothenic acid, and riboflavin). We demonstrate that benthic organisms are sources of complex mixtures of metabolites that vary by species and that seawater microorganisms respond differently to exudates from stony corals and octocorals.

## Materials and methods

### Benthic organism exudate collections

Exudate collections from benthic organisms were conducted on board the R/V *Walton Smith* in November 2018 in Lameshur Bay, St. John, U.S. Virgin Islands within the Virgin Islands National Park. In brief, we collected six species of benthic organisms (*n* = 6 specimens), incubated these organisms in separate containers for 8 h, and harvested the incubation water to characterize the composition of dissolved metabolites in their exudates. A description of the exudate collections is included below (additional details available in Supplementary Methods).

Before each organism experiment, 58 l of surface (non-reef) seawater was collected ~1 mile offshore (18 17.127° N, 064 44.312° W, 31.6 m depth). Cells and particles were removed using peristaltic pressure through a 0.2 µm filter (47 mm, Omnipore, EMD Millipore Corporation, Billerica, MA, USA) using metabolomics-grade tubing and this filtrate (filtered seawater) was collected for the incubations. Additionally, two to three, 2 l filtrate subsets per experiment were acidified with concentrated hydrochloric acid (final concentration 1% volume/volume) and subjected to solid-phase-extraction (SPE) using a negative vacuum pressure of –3.7 to –5 100xkPA in Hg, to serve as controls. Before SPE, 6 ml, 1 gm Bond Elut PPL cartridges (Agilent, Santa Clara, CA, USA) were pre-conditioned with 6 ml of 100% HPLC-grade methanol.

For the experiments, six species of benthic organisms were collected from reefs around Lameshur Bay by SCUBA divers. Experiments were completed on three stony corals (*Porites astreoides*, *Siderastrea siderea*, and *Psuedodiploria strigosa*), two octocorals (*Plexaura homomalla* and *Gorgonia ventalina*), and one encrusting alga (*Ramicrusta textilis*) (Table S1). *P. astreoides*, *S. siderea*, and *R. textilis* were held in a seawater table for 24 h (hrs) before the incubations and colonies from the other three species were held for 2-3 h due to timing constraints. Coral and algal fragments were generally small (2.5-5.0 cm in length).

For each incubation, nine, acid-washed, 10 l polycarbonate bins (with lids) containing filtered seawater (4 l) were secured into an illuminated aquarium table (Prime HD, Aqua illumination, Bethlehem, PA, USA) (Photosynthetically Active Radiation = ~350–600 µmol quanta m^−2^ s^−1^). Air bubblers with sterilized Fluorinated Ethylene Propylene (FEP) tubing (890 Tubing, Nalgene, Thermo Scientific, Waltham, MA, USA) were used to inject air into each bin. Surface seawater was circulated through the aquarium table to maintain reef seawater temperature (29.5 °C). Six colonies/fragments of one species were randomly placed into 6 bins. The other 3 bins were reserved for control incubations containing filtered seawater only. A sensor (8 K HOBO/PAR loggers; Onset, Wareham, MA) monitored temperature and light conditions (data not shown). At the end of each 8 h experiment, colonies/fragments were wrapped in combusted aluminum foil and flash frozen in a charged dry shipper. The water in all incubations was re-filtered (as outlined above) and 2 l of each filtrate were acidified and subjected to SPE as described above. SPE cartridges were wrapped in combusted aluminum foil, placed in Whirl-Pak (Nasco, Madison, WI, USA) bags, and frozen at –20 °C.

### Metabolomics analyses and data processing

At the Woods Hole Oceanographic Institution (WHOI), metabolites were eluted from the thawed cartridges into combusted, borosilicate test tubes using 100% methanol (Optima grade) within 3 months of collection. The eluents were transferred into combusted amber 8 ml vials and nearly dried using a vacuum centrifuge. Samples were reconstituted in 200 µL of 95:5 (v/v) Milli-Q (MQ, Millipore Sigma, Burlington, MA, USA) water: acetonitrile with a deuterated standard mix added as an internal control (Table S2), vortexed, and prepared for targeted and untargeted metabolomics analyses in both positive and negative ion modes as described previously [16]. Samples prepared for untargeted analyses were further diluted (1:200) with the reconstitution solvent. A pooled sample (technical replicate) was made by combining aliquots from all samples and was injected repeatedly to assess instrument drift over the course of the run and for downstream sample processing. Samples prepared for targeted metabolomics were analyzed using an ultra-high performance liquid chromatography system (UHPLC; Accela Open Autosampler and Accela 1250 Pump, Thermo Scientific, Waltham, MA, USA) coupled to a heated electrospray ionization source (H-ESI) and a triple stage quadrupole mass spectrometer (TSQ Vantage, Thermo Scientific), operated in selected reaction monitoring (SRM) mode. Samples prepared for untargeted metabolomics were analyzed with a UHPLC system (Vanquish UHPLC, Thermo Scientific) coupled to an ultra-high resolution mass spectrometer (Orbitrap Fusion Lumos, Thermo Scientific). MS/MS spectra were collected in a data-dependent manner using higher energy collisional dissociation (HCD) with a normalized collision energy of 35% (detailed methods provided in [16]). A Waters Acquity HSS T3 column (2.1 × 100 mm, 1.8 μm) equipped with a Vanguard pre-column was used for chromatographic separation at 40 °C for targeted and untargeted analyses. Sample order was randomized and the pooled sample was analyzed after every six samples.

For targeted metabolomics analysis, tandem MS/MS data files were converted into .mzML files using msconvert and processed with El-MAVEN [49]. Calibration curves for each compound (8 points each) were constructed based on the integrated peak areas using El-MAVEN. The concentrations of metabolites in the original samples were determined by dividing each concentration by the volume of the filtrate that passed through each PPL column. Finally, metabolite concentrations above the limits of detection and quantification were corrected for extraction efficiency using in-house values determined using standard protocols [50]. Statistical analyses of targeted metabolite concentrations were conducted using Welch’s independent t-tests and ANOVAs or Wilcoxon rank sum tests if data were not normally distributed (additional details in Supplementary Methods). We determined the mass of each colony and conducted Pearson correlations to investigate if colony size significantly correlated with concentrations of targeted metabolites, but no correlations were found.

For the untargeted metabolomics analyses, raw files containing MS1 and MS/MS data were converted into .mzML files using msconvert and processed using XCMS [51]. Ion modes were analyzed separately. Before processing with XCMS, the R package AutoTuner [52] was used to find XCMS processing parameters appropriate for the data. In XCMS, the CentWave algorithm picked peaks using a gaussian fit. The specific parameters for peak picking for both ion modes were: noise = 10,000, peak-width = 3–15, ppm = 15, prefilter = c(2,168.600), integrate = 2, mzdiff = –0.005, snthresh = 10. Obiwarp was used to adjust retention times and this step was followed by correspondence analysis. For statistical analyses, including permutational PERMANOVA adonis tests and non-metric multidimensional scaling analysis (NMDS), MS1 features (defined as unique pairings of mass-to-charge (*m/z*) values with retention times) in both ion modes were culled following XCMS if they: (1) had >1 average fold change in the MQ blanks compared to the other samples, (2) occurred in less than 20% of samples (excluding pooled controls), and/or (3) were invariant (relative standard deviation of <15%) across the samples [53]. This feature-removal strategy retained 27% (2317) of MS1 features ionized in positive mode and 84% (4725) of features ionized in negative mode. The R packages ggplot2 [54] and vegan [55] were used to visualize trends and inspect compositional differences among samples in the untargeted datasets using Bray-Curtis dissimilarity, non-metric multidimensional scaling analysis, and permutational PERMANOVA (adonis) tests with and without constraining permutations across the different incubations. Non-Euclidean NMDS ordination and permutational multivariate analysis of variance (adonis) methods were used because these approaches are well-suited for investigating compositional dissimilarities among samples in sparse, semi-quantitative datasets.

The MetaboAnalyst 5.0 web browser [56] was used to putatively identify enriched metabolic pathways in the untargeted MS1 features ionized in positive mode resulting from the XCMS processing (as described above) using the mummichog algorithm [57]. Prior to analysis, zero or missing feature intensities were replaced by the 1/5 minimum positive feature intensity and feature intensities were log_10_-normalized. Enrichment analysis of metabolic pathways was conducted by comparing control samples containing no organisms to organism incubation samples (all species). Additional details are reported in Supplementary Methods.

The .mzML files containing MS1 and MS/MS spectra from untargeted data analyzed in positive ion mode were interrogated using classical molecular networking (version release 28.1) [58] available through the Global Natural Products Social Molecular Networking (GNPS) database. Classical molecular networking was conducted using default parameters (pre-cursor ion mass tolerance = 2.0 Da, fragment ion mass tolerance = 0.5 Da, minimum cosine pair = 0.7, minimum matched fragment ions = 6) and the results can be viewed using the following link: https://gnps.ucsd.edu/ProteoSAFe/status.jsp?task=8abc6ce7fd334ff19eee1d0df12dcfe0. The average number of input MS1 features for classical molecular networking was 1014 with 4748 MS/MS spectra per sample. We also tested feature based molecular networking (version release 28) [59] using filtered MS1 data for both positive and negative ion modes. Raw and derived (.mzML) files from targeted and derived files from untargeted metabolomics analyses can be accessed in the MetaboLights database under accession MTBLS2855.

### Exudate uptake experiments

To assess the lability of organism exudates, we examined the responses of reef seawater microbial communities to exudates from *P. astreoides* and *G. ventalina*. After the organism incubations described above, excess filtrate (2 l) from 3 of the 6 organism incubations (selected randomly) was pooled into an acid-washed 10 l carboy, and excess filtrate (~2 l) from the three control incubations was pooled into a second, acid-washed 10 l carboy.

For the *P. astreoides* experiment, surface seawater was collected from the offshore site and coarsely filtered through a GF/A filter (1.6 µm nominal pore size) using peristalsis to remove larger cells and minimize heterotrophic grazing, while retaining bacteria and archaea. Approximately 2.4 l of this inoculum was added separately to each 10 l carboy of either pooled coral or control filtrate to create a 5:2 ratio of filtrate: inoculum. After this addition, each carboy was mixed and a suite of samples were collected for different analyses including cell abundance enumeration, inorganic and organic macronutrient quantification, and 16 S rRNA gene sequencing. This initial collection was the first time point (0 h) for the exudate uptake experiment. For the *G. ventalina* experiment, reef seawater inoculum was collected from Tektite reef (Table S1).

For each experiment, coral metabolite or control filtrate seawater was transferred into 1 l acid-washed polycarbonate bottles (6 bottles per treatment). Within each treatment (coral and control), 3 of the 6 bottles were blackened to block light. The bottles were placed into a flow-through seawater table. PAR readings in the seawater table for the *P. astreoides* and *G. ventalina* experiments were 250–1000 and 164–530 µmol quanta m^−2^ s^−1^, respectively, with variation caused by changes in cloud cover. Over 48 h, samples were collected for cell enumeration (1 ml) at all time points (0, 12, 24, 36, and 48 h), macronutrient analyses (30–40 ml) at 0 and 48 h, and microbial community analyses (60–300 ml) at 0, 24, and 48 h.

Samples collected for cell enumeration were fixed to 1% (v/v) paraformaldehyde, refrigerated for 20 minutes in the dark, and frozen in a charged dry shipper. Abundances of *Prochlorococcus*, *Synechococcus*, picoeukaryotes, and unpigmented cells were enumerated via flow cytometry (Supplementary Methods). Cell abundances collected at 12, 24, 36, and 48 h were normalized using initial cell abundances at 0 h.

Samples (40 ml) were collected for total organic carbon (TOC) and total nitrogen (TN) analyses into combusted, borosilicate EPA vials and acidified using 75 µl of concentrated phosphoric acid. Samples were stored at room temperature for two weeks and then refrigerated at 4 °C until analysis. Samples were analyzed at WHOI using a Shimadzu TOC-V_CSH_ total organic carbon analyzer with a TNM-1 module [60].

For inorganic nutrient analyses, seawater (30 ml) was allocated into acid-washed, polypropylene bottles (Nalgene) and frozen at −20 °C. These samples were shipped to Oregon State University and the concentrations of nitrite, nitrite + nitrate, ammonium, silicate, and phosphate were obtained using a continuous segmented flow system (described in [61]). Total organic nitrogen concentrations were obtained by subtracting the sum of the inorganic nitrogen species (nitrite + nitrate and ammonium) from the total nitrogen concentrations. Values measured below the detection limits of the instruments (ammonium = 0.02 μM, phosphate = 0.01 μM, nitrite + nitrate = 0.07 μM, nitrite = 0.01 μM) were reported as zero. Cell abundances and nutrient concentrations can be accessed via the Biological and Chemical Oceanography Data Management Office (BCO-DMO) under dataset accessions 865739 and 865193, respectively.

Seawater samples (60 ml for *P. astreoides*) collected for microbial community analyses were obtained using 60 ml sterile, Luer-lock syringes (Becton Dickinson, Franklin Lakes, NJ, USA) and filtered using positive pressure onto 25 mm, 0.2 μm pore-size filters. The amount of volume filtered was increased for the *G. ventalina* exudate experiment because of biomass concerns and ranged from 180 (0 and 24 h) to 300 ml (48 h). Previous work has demonstrated that sample volume does not significantly influence microbial community composition when beta diversity comparisons are made [24]. Alpha diversity analysis was avoided due to the differences in seawater volumes. Filters were then transferred into cryovials and placed in the charged dry shipper, followed by storage at –80 °C, until DNA extraction.

DNA was extracted from filters using the Qiagen PowerBiofilm extraction kit (Qiagen, Germantown, MD, USA) following default instructions. Five DNA extraction controls were created alongside the samples by performing the extractions without filter biomass. Purified DNA from a mock community (BEI Resources, NIAID, NIH as part of the Human Microbiome Project: Genomic DNA from Microbial Mock Community B (Even, Low Concentration), v5.1 L, for 16 S rRNA Gene Sequencing, HM-782D) was included in the sequencing library to assess PCR performance. Samples were amplified using the primers 515F-Y [62] and 806R-B [63] with conditions outlined in the Supplementary Methods and sequenced using a benchtop iSeq 100 sequencer (Illumina, San Diego, CA, USA). Two sequencing libraries were built to maximize sequence coverage per sample.

Across both sequenced libraries, the average number of initial reads in non-control samples was 139,508 ± 55,490 (standard deviation). To analyze the sequencing data, all the demultiplexed fastq files across both libraries were compiled and run together using the DADA2 R package [64]. Reverse reads were dropped from the analysis due to lower read quality and concatenation difficulties due to the shorter reads. Based on previous work comparing phylogenetic resolution from different lengths of V4 reads [65], our general taxonomic results are likely comparable between the iSeq and MiSeq-based (described below) results. Forward reads were then filtered using the ‘filterAndTrim’ parameter (trimLeft = 19, truncLen = 145, maxN = 0, maxEE = 1, rm.phix = TRUE). Prior to inferring amplicon sequence variants (ASVs), error rates were obtained and screened. Chimeras were removed (~2% of all remaining sequences after the filtering), and the average number of reads per sample after the filtering, denoising, and chimera removal steps dropped to 123,867 (14,703–281,252 range). The average number of reads in the 5 DNA extraction control samples was 3351(1092–6600 range) and the negative PCR control had 1471 reads. Taxonomy was assigned using the Silva v.138 database [66] and the “addSpecies” function was used to assign more specific taxonomy designations, resulting in 255 species-level assignments out of 7364 ASVs. Mock community performance was also assessed, and 43 and 26 ASVs were detected in the sequenced mock community samples. All ASVs (consisting of forward reads only) detected in the mock communities were continuous partial matches to the longer sequence reads included in the mock reference file. The R package decontam [67] was used to remove contaminating sequences from the samples using either their prevalence or frequency in the 5 DNA extraction controls, decreasing the number of ASVs from 7364 to 7333. Samples with <7000 sequences and sequences identifying as NA or uncharacterized at the phylum level were removed. Sequences identifying as chloroplasts at the order level were also removed. After conducting these filtering steps, we detected an average of 388 ASVs (standard deviation ±227 ASVs) across control and coral exudate addition treatments.

The R package corncob [68] was used to identify ASVs that significantly covaried based on differential abundance with treatment type (coral exudate addition vs. control) at each individual time point across the two experiments. Corncob was run using the Wald test with an FDR cut-off of 0.05. Sample counts were transformed to relative abundance for taxonomic comparisons and boxplots were generated to verify the corncob results. To summarize these results, ASVs were selected that displayed consistent patterns of increase or decrease in relative abundance over time. In addition, only ASVs with coefficients of variation less than –2 (depleted in coral exudate additions) and greater than 0 (enriched in coral exudate additions) were included in this summary analysis to focus on ASVs displaying the most distinct changes. The relative abundance of each ASV was averaged across replicates and normalized to the initial abundance of that ASV at the first time point (0 h). Using these averages, fold changes (coral addition/control) were calculated for each ASV and the log_10_ of the fold changes were plotted. To prevent the formation of infinite values, relative abundances of 0 were substituted with low values (1e-7). Non-metric multidimensional scaling was completed on the Bray-Curtis dissimilarity matrix to compare overall compositional differences between samples. Adonis tests were completed on the Bray-Curtis dissimilarity matrix to test which factors significantly influenced dissimilarity between samples. The raw fastq files containing sequences for these samples can be accessed on the NCBI Sequence Read Archive (SRA) under BioProject PRJNA739882.

### Metabolite uptake incubations

Three metabolite uptake incubation experiments were conducted in St. John, USVI in January 2021 based on the results of the benthic organism incubations. These experiments assessed if reef seawater microbial communities would respond distinctly to different metabolites. Experiments were conducted with three metabolites (riboflavin, pantothenic acid, and caffeine) in separate incubations occurring on different days. The B vitamins riboflavin and pantothenic acid were chosen because they were released by at least 4 of 6 species during our benthic organism incubations. Caffeine was also selected because it was released in high quantities by the invasive alga *R. textilis*. All three metabolites were also detected and quantified in seawater collected from reefs in the Jardines de la Reina reef-system in Cuba [16]. Samples were collected at three time points over 24 h: 0, 6, and 24 h. Incubations were conducted in the dark and processed in low-light conditions to minimize photodegradation of the metabolites, especially riboflavin.

Metabolite standards were diluted with MQ water to 5 nM (riboflavin, pantothenic acid) and 10 nM (caffeine) one month prior to the incubations, frozen, and shipped to the USVI. Metabolite dilutions were prepared in combusted amber vials and kept in the dark to minimize photochemical degradation. To set up the incubations, 5.5 l of seawater was collected approximately 1 m above Tektite reef (9 m depth) using Niskin bottles deployed by divers. Half of the seawater was filtered using a 0.1 µm, 47 mm Omnipore filter to create filtered seawater, while the rest of the seawater was filtered using a 1 µm, 47 mm Omnipore filter to remove larger phytoplankton and protistan grazers but retain picoplankton. The filtrate treatments were poured into 36 acid-washed and autoclaved 125 ml polycarbonate bottles. To account for small volumes and minimize the chance of sample contamination, incubations were designed so that bottles could be sampled by sacrifice at each time point. Each incubation had four different experimental conditions: 3 different controls and one experimental treatment. The three controls included filtered seawater (F), filtered seawater with the addition of a metabolite spike (F + Mtb), and 1 µm filtered seawater containing microbes (M). The experimental condition was 1 µm filtered seawater containing microbes with the addition of the metabolite (M + Mtb). Metabolite spikes (20 pM for riboflavin and pantothenic acid and 70 pM for caffeine) were added to the F + Mtb and M + Mtb treatments by removing 500 µl (riboflavin and pantothenic acid incubations) or 875 µl (caffeine incubation) of seawater from each incubation bottle, replacing the lost volume with the appropriate metabolite spike, and inverting the bottles to mix. After the metabolite spikes were added, 24 of 36 incubation bottles were placed in a flow-through seawater table covered with a tarp and equipped with a HOBO data logger to monitor relative light levels and water temperature (data not shown), while the remaining 12 bottles, accounting for triplicate bottles of each of the four experimental conditions, were immediately processed for the initial time point.

After each time point, samples from the incubation bottles were re-filtered using 0.2 µm, 47 mm Omnipore filters and filtrate was collected and acidified with 125 µl of concentrated hydrochloric acid. The filters were placed into cryovials and frozen in a dry shipper. All samples were filtered and acidified within one hour of collection. SPE was conducted on filtrate using the methods outlined above. Bottle masses were obtained prior to and after SPE to account for sample volume. Additionally, subsamples of the initial and final incubation samples were obtained to assess microbial cell counts across the different treatments using flow cytometry (described in Supplementary Methods).

### Metabolite uptake incubation data processing and analyses

The previously frozen PPL cartridges were eluted using 6 ml of 100% methanol within 3 weeks of sample collection and samples were prepared for targeted metabolomics analysis in positive ionization mode. Samples were dried, re-suspended in 200 µl of 50–150 ng deuterated standard mix dissolved in MQ water (Table S2), vortexed, and aliquoted into pre-combusted analysis vials containing inserts. A pooled sample was created for instrument quality control and to make a matrix-matched standard curve. To make the 11-point matrix-matched standard curve, 50 µl of pooled sample was combined with MQ water and various concentrations (ranging from 0.5 to 1000 ng ml^-^1) of metabolite mixes prepared in MQ water. Instrument conditions and data processing were identical to the methods outlined above. The targeted raw and .mzML files can be accessed on the MetaboLights database under accession MTBLS3286.

To generate the sequencing library for microbial community analysis, DNA was extracted from filters using the PowerBiofilm DNA extraction kit following the standard protocol. Seven DNA extraction controls were created to test for contamination. Amplification was conducted with the 515F-Y [62] and 806R-B [63] primers and sequencing occurred using Illumina MiSeq 2×250 bp, with additional details recorded in Supplementary Methods.

Trends in microbial abundances of *Prochlorococcus*, *Synechococcus*, picoeukaryotes, and unpigmented cells (~heterotrophic bacteria and archaea) yielded via flow cytometry were inspected using line graphs and standard error was calculated across replicate samples for each time point. Microbial community analysis was completed using the methods outlined above for the exudate uptake experiments including general compositional analysis as well as specific ASV enrichment analysis using corncob. An average of 232 ASVs (standard deviation ± 135) per sample were recovered. The data is available on NCBI Sequence Read Archive (SRA) under BioProject PRJNA739882. Cell abundances can be accessed via BCO-DMO under the project entitled “Signature exometabolomes of Caribbean corals and influences on reef picoplankton” (dataset accession 865159).

## Results

### Metabolites released during benthic organism incubations

**Fig. 1:**
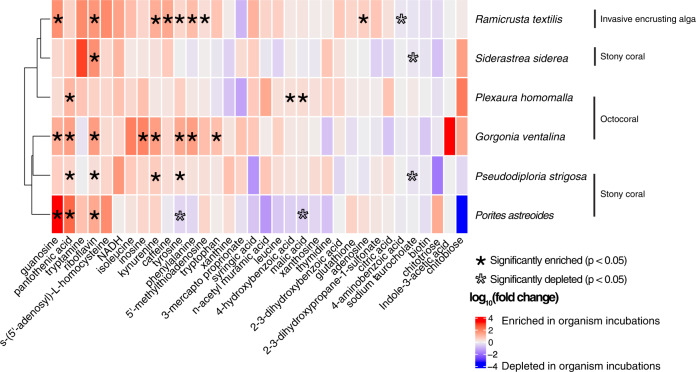
Heatmap of average log_10_(fold changes) between organism and control incubations demonstrates that specific targeted metabolites are significantly enriched and depleted and vary by species. Colors correspond to log_10_(fold change) and asterisks reflect significantly enriched (black) or depleted (outlined) metabolites in organism compared to control incubations as determined using Welch’s t-tests.

Using targeted metabolomics, we detected 48 metabolites and quantified 34 of 48 metabolites in incubation seawater (Table S3). Exudates from the invasive alga *R. textilis* had the highest number of significantly enriched metabolites compared to control incubation water containing no benthic organisms (*p* < 0.05, Welch’s *t* test), followed by exudates from *G. ventalina* (Fig. 1). Metabolites generally displayed differing trends by species, but guanosine, pantothenic acid, riboflavin, and kynurenine were consistently enriched in organism compared to control incubations (Fig. 1 and Fig. S1). Tyrosine was significantly enriched in exudates from *R. textilis*, *G. ventalina*, and *P. strigosa*, but depleted in *P. astreoides* incubations. Caffeine was only significantly enriched in *R. textilis* exudates. Exudates from *S. siderea* and *P. homomalla* were most like the controls. The fold change of tryptamine was high in *S. siderea* incubations, but not significant. Similarly, indole-3-acetic acid was only detected in five of the six *G. ventalina* incubations (Fig. S2) but was not significantly enriched. Some metabolites exhibited variable concentrations across incubations and control replicates (Table S3). To examine potential experimental artifacts due to incubation conditions, metabolite concentrations were compared between control incubations containing no benthic organisms and freshly collected seawater and were generally similar except for 5’-methylthioadenosine, NADH, adenosine, glutathione (oxidized), kynurenine, and riboflavin (ANOVA, *p* < 0.05). Concentrations of NADH and riboflavin were significantly higher in freshly collected seawater whereas concentrations of 5’-methylthioadenosine, adenosine, glutathione, and kynurenine were significantly higher in control incubations containing no benthic organisms (*p* < 0.05, Welch’s *t* test) (Table S3).

**Fig. 2:**
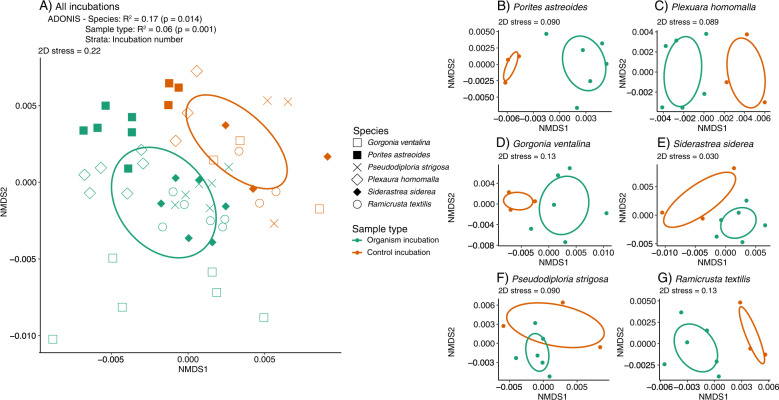
Non-metric multidimensional scaling analysis (NMDS) of Bray-Curtis dissimilarities calculated for untargeted metabolite features ionized in positive mode are significantly influenced by species and sample type (organism vs. control incubations). **A** Global NMDS of control and organism incubations is displayed for all species and the results of the constrained adonis test are included above the plot. **B**–**G** NMDSs of control and organism incubations are displayed separately for each coral species. Symbol shape corresponds to species and color corresponds to sample type. Ellipse shapes indicate covariances among samples in each grouping.

Permutational adonis tests revealed that metabolite composition via the untargeted method was significantly influenced by species, sample type (organism vs. control), and incubation in both ion modes (Fig. 2, Table S4). Furthermore, species and sample type remained significant when drift across incubations was ignored by constraining permutations. Non-metric multidimensional scaling analysis revealed that untargeted feature compositions were similar between positive (Fig. 2A) and negative ion modes (Fig. S3). Organism and control feature compositions were distinct from each other (Fig. 2 and Fig. S3). Organism feature compositions generally grouped by species, but there was more overlap across *P. strigosa*, *S. siderea*, and *R. textilis* feature compositions (Fig. 2 and Fig. S3). Exudates harvested from *G. ventalina* and *P. astreoides* plotted outside of the covariance matrix for the incubations, indicating that these exudates were more dissimilar from exudates released by the other benthic organisms (Fig. 2A). Differences in metabolite composition were more distinct in positive compared to negative mode; there was more overlap between control and organism incubation samples for *R. textilis*, *P. astreoides*, and *P. strigosa* in negative mode (Fig. S3). Feature compositions ordinated by sample type when species were plotted separately (Fig. 2B–G). Reflecting the targeted data, non-incubation seawater and control samples containing no benthic organisms were similar in untargeted feature compositions across both ion modes (Fig. 2A and Fig. S3).

We used the MetaboAnalyst 5.0 web-based browser [56] to further interrogate the untargeted features ionized in positive mode and found that the chemical classes of indoles and ketones were significantly enriched (gamma *p*-values < 0.05) in organism incubations compared to control incubations containing no benthic organisms (Table S5). Classical molecular networking of untargeted features ionized in positive mode in GNPS [58] yielded 7 unique feature matches out of 11 total matches. Upon further inspection, we were not able to validate the matches due to large *m/z* discrepancies (additional details in Supplementary Methods).

### Microbial responses to coral exudates

**Fig. 3:**
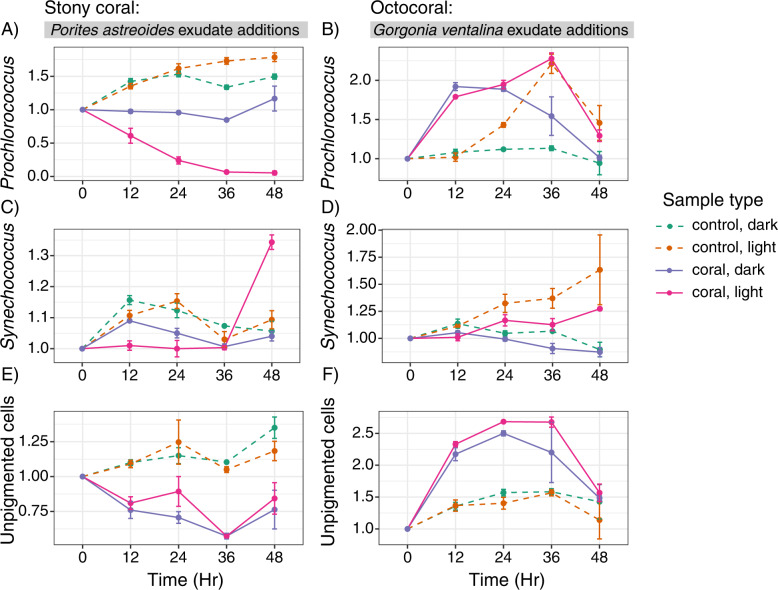
Abundances of *Prochlorococcus*, *Synechococcus*, and unpigmented cells respond differently to exudates from *Porites astreoides* and *Gorgonia ventalina*. Line graphs of normalized cell counts of *Prochlorococcus* (**A**, **B**), *Synechococcus* (**C**, **D**), and unpigmented cells (**E**, **F**) show differing responses of microbial communities to *Porites astreoides* (**A**, **C**, **E**) and *Gorgonia ventalina* (**B**, **D**, **F**) exudates. Color corresponds to sample type, symbols represent average cell counts across replicates, and error bars reflect standard error across replicates (*n* = 3 per treatment type). Dashed lines represent controls and solid lines represent coral exudate addition treatments.

Exudates from the stony coral *P. astreoides* and octocoral *G. ventalina*, which showed distinctly different metabolite signatures (Figs. 1 and 2A), elicited differential responses in microbial abundances (Fig. 3). *P. astreoides* exudates inhibited the growth of *Prochlorococcus* in light relative to the dark treatments and compared to controls that contained no coral exudates (Fig. 3A). Conversely, *G. ventalina* exudates enhanced the growth of *Prochlorococcus* in both light and dark conditions until 36 h (Fig. 3B). *Synechococcus* abundances remained relatively low in the light, with *P. astreoides* exudates causing 1.3x enhancement during 36–48 h (Fig. 3C). However, in the presence of *G. ventalina* exudates, *Synechococcus* abundances were highest in control samples containing no coral exudates (Fig. 3D). The abundance of unpigmented cells (primarily heterotrophic bacteria and archaea) differed between the two exudate additions, with cells plummeting relative to controls upon *P. astreoides* exudate exposure (Fig. 3E) and cells nearly doubling by 12 h upon *G. ventalina* exudate exposure and growing further over 36 h (Fig. 3F). There were no consistent patterns for picoeukaryotic cell populations (data not shown).

**Fig. 4:**
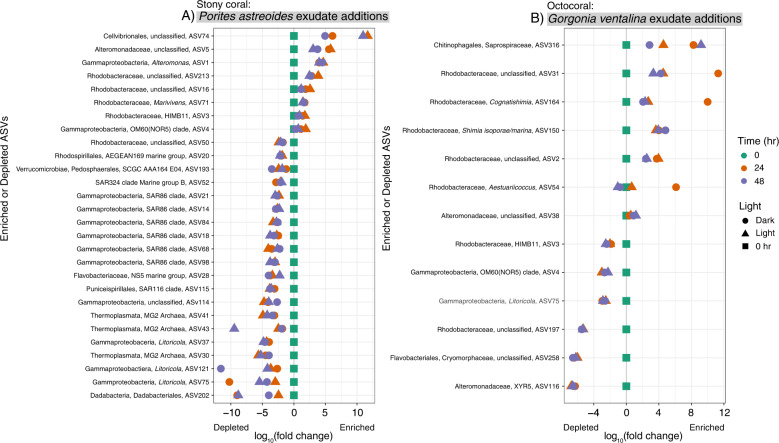
Relative abundances of specific microbial taxa increase or decrease in the presence of *Porites astreoides* and *Gorgonia ventalina* exudates. Log_10_(fold change) plots of averaged microbial 16 S rRNA gene ASV relative abundances (normalized to the initial time point at 0 h) that significantly and consistently increased or decreased in the presence or absence of *Porites astreoides* (**A**) and *Gorgonia ventalina* (**B**) exudates determined using corncob analysis. Only ASVs with coefficients of variation less than –2 (depleted in coral exudate additions) and greater than 0 (enriched in coral exudate additions) were included to focus on ASVs displaying the most distinct changes. Symbol color reflects the time that samples were collected during the incubations and shape reflects light conditions.

The addition of exudates from *P. astreoides* and *G. ventalina* led to consistent enrichment or depletion of bacterial and archaeal taxa grouped at the level of amplicon sequence variants (ASVs) relative to controls containing no coral exudates at 24 and 48 h and impacted overall microbial community composition (Fig. 4, Figs. S4 and S5). Additionally, exudate source prompted differential responses in microbial taxa. *P. astreoides* exudates led to higher relative abundances of 8 different ASVs including various unclassified Rhodobacteraceae, OM60(NOR5) clade, *Marivivens*, *Roseobacter* HIMB11, unclassified Cellvibrionales, *Alteromonas*, and unclassified Alteromonadaceae (Fig. 4A). Interestingly, *P. astreoides* exudates also led to the depletion of 20 ASVs relative to controls. Most of the depleted ASVs identified as Gammaproteobacteria within the SAR86 clade (7 ASVs), *Litoricola* (3 ASVs), and Marine Group II Archaea (3 ASVs).

**Fig. 5:**
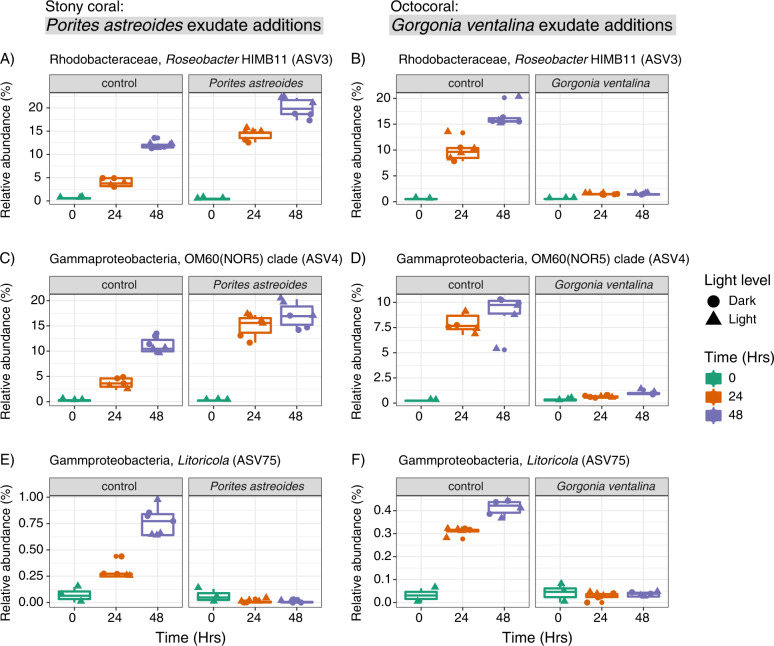
Relative abundances of specific microbial taxa exhibit similar or different responses to exudates from *Porites astreoides* and *Gorgonia ventalina* overtime. Boxplots display relative abundances of ASVs that exhibit different (**A**–**D**) or similar (**E**, **F**) trends in response to coral exudates. Plots (**A**, **C**, and **E**) show trends in response to *P. astreoides* exudates and plots (**B**, **D**, and **F**) show trends in response to *G. ventalina* exudates. Symbol color indicates time and shape reflects light conditions.

Exudates from *G. ventalina* led to increases in the relative abundances of 10 ASVs (Fig. 4B). Most of the enriched ASVs classified as Rhodobacteraceae (unclassified Rhodobacteraceae, *Shimia isoporae*, *Cognatishimia, Aestuariicoccus)* and Alteromonadaceae. In contrast to the *P. astreoides* exudate additions, relative abundances of HIMB11(ASV3) and OM60(NOR5) (ASV4) were depleted in *G. ventalina* exudate additions (Fig. 5). Relative abundances of *Litoricola* (ASV75) decreased in the presence of *G. ventalina* exudates and echoed the trends of this ASV in the presence of *P. astreoides* exudates (Fig. 5). Additionally, the relative abundance of *Alteromonas* constituted most of the microbial community at 24 and 36 h after the beginning of the experiment in the presence of *G. ventalina* exudates (Fig. S5).

Across the two incubations, microbial community composition did not differ between samples at the initial time point (0 h), but treatment (coral vs. control), exudate source (*P. astreoides* vs. *G. ventalina*), light exposure, and time were significant (*p* < 0.001) drivers of microbial community Bray-Curtis dissimilarity as revealed by an adonis test. Exudate source had the highest R^2^ value of 0.24, indicating that exudate source/composition was the most significant factor influencing microbial community dissimilarity over time. In fact, exudate source remained significant when the treatment factor was ignored using constrained permutations. Along with changes in microbial abundances and community composition, organic and inorganic nutrient concentrations varied over time and in relation to exudate source, but there were no consistent trends (Figs. S6–S8).

### Microbial responses to metabolite additions

**Fig. 6:**
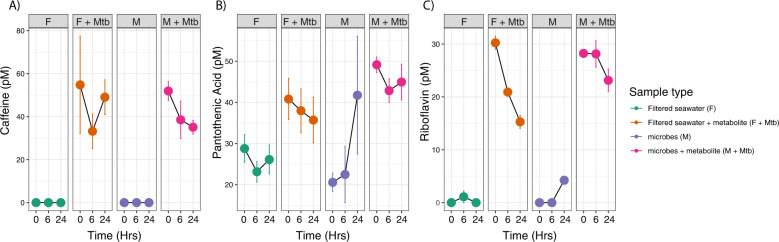
Concentrations of caffeine, pantothenic acid, and riboflavin exhibit different trends in the presence or absence of reef seawater microbial communities overtime. Variations in **A** caffeine, **B** pantothenic acid, and **C** riboflavin concentrations overtime show that metabolites experience different trends in the presence/absence of reef seawater microbial communities. Color corresponds to sample type, symbols represent average cell counts across replicates, and error bars reflect standard error computed across replicates.

Metabolites of interest from the organism incubations, including caffeine, pantothenic acid, or riboflavin, were added to reef seawater incubations, with microbes (M + Mtb treatments) and without microbes (F + Mtb treatments). The individual metabolites were tracked for 24 h to monitor microbial changes. All metabolites were detected in the incubation bottles to which they were added and initial measured and expected concentrations were similar (maximum error of 1.25%) (Fig. 6). Background concentrations of pantothenic acid in F and M treatments ranged from 20 to 30 pM (Fig. 6B), but initial background concentrations of caffeine and riboflavin were very low or undetectable (Fig. 6A, C). Caffeine concentrations decreased in the M + Mtb treatment over the course of 24 h but remained similar in the F + Mtb treatment (Fig. 6A). Caffeine concentrations were undetectable in both F and M treatments throughout the incubation. Pantothenic acid concentrations rapidly increased 6 to 24 h after the beginning of the incubation in the M treatment, notably reaching concentrations similar to the M + Mtb treatment by the end of the incubation (Fig. 6B). Pantothenic acid concentrations hovered around ~25.6 pM in the F treatment and average concentrations decreased slightly in the F + Mtb treatment. Riboflavin concentrations were mostly undetectable in the F treatment and low in the M treatment until 24 h after the start of the incubation when the concentration increased to 4.23 pM (Fig. 6C). Riboflavin concentrations decreased consistently in the F + Mtb treatment with an average rate of decrease of 0.91 pM/hour (0–6 h: 1.5 pM, 6–24 h: 0.312 pM). In the M + Mtb treatment, riboflavin concentrations held steady at ~28 pM after 6 h, but decreased slightly to 23.13 pM at 24 h. Interestingly, microbial abundances (Fig. S9) and microbial community composition (Fig. S10) did not respond strongly or consistently to the added metabolites across all three incubations, but did change over time.

## Discussion

Our study presents an extensive profile of exudates from benthic reef organisms and places this knowledge into an ecological context by monitoring the responses of reef microbial communities to complex mixtures of exudates from two species of corals and to individual metabolites that emerged as important in the coral and algal exudates. Overall, these results demonstrate that exudates from benthic organisms contribute to the complex pool of extracellular metabolites in reef seawater and that exudate composition varies significantly by species. We found that Caribbean stony coral and octocoral exudates differentially impact abundances and compositions of reef microorganisms and that complex exudate mixtures, rather than the individual metabolites (riboflavin, caffeine, and pantothenic acid), select for specific microbial taxa. This work has important implications for understanding microbially-mediated benthic-pelagic coupling within threatened Caribbean reefs.

### Responses of marine microorganisms to exudates

Reef seawater microbial communities differentially responded to exudates released from *P. astreoides* and *G. ventalina*, consistent with results from other studies [30–32]. We also show that exudates are species-specific, aligning with previous results that found coral and algal exometabolites to be distinct from each other [33], and that exudate mixtures from different corals select for distinctive microbial communities. In our study, exudates from *P. astreoides* inhibited the growth of *Prochlorococcus* and unpigmented cells while exudates from *G. ventalina* stimulated the growth of these groups. In fact, *P. astreoides* exudates depressed *Prochlorococcus* cell abundances in the presence of light, indicating that *Prochlorococcus* may be sensitive to the interactive effects of light and specific metabolite mixtures, although the mechanisms behind this response are unknown. *G. ventalina* exudates exhibited a rapid stimulatory response in *Prochlorococcus* in both the dark and light treatments within 24 h, suggesting that *G. ventalina* metabolites hasten cell division in *Prochlorococcus* more so than just the presence of light alone.

Exudates from *P. astreoides* consistently enriched for specific ASVs identified as unclassified Rhodobacteraceae, gammaproteobacteria within the OM60(NOR5) clade, other unclassified gammaproteobacteria within the order Cellvibrionales, as well as *Marivivens*, Roseobacter HIMB11, *Alteromonas*, and unclassified Alteromonadaceae. Exudates from *G. ventalina* enriched for more ASVs, including Rhodobacteraceae (*Shimia isoporae*, *Cognatishimia*, *Aestuariicoccus* and unclassifieds), and Alteromonadaceae, but did not stimulate Roseobacter HIMB11 or OM60(NOR5). Many of these taxa are typically present at low relative abundances in reef seawater [69], detected in seawater from coastal, in-shore reefs [70], and associated with coral and algal exudates [31]. Enrichment of Rhodobacteraceae has been observed previously in the presence of *P. astreoides* exudates in mesocosm-based experiments [71] and there is evidence that Rhodobacteraceae in reef seawater exhibit chemotaxis towards mixtures of metabolites, including amino acids [72]. Furthermore, relative abundances of Rhodobacteraceae generally correlate with nutrient availability on reefs [70], indicating the more copiotrophic nature of these bacteria. Various gammaproteobacteria, including *Alteromonas*, were significantly enriched in seawater immediately surrounding different species of corals, indicating that these taxa are associated with coral exudates [24]. Additionally, *Alteromonas* strains have been isolated from coral mucus and skeletons [73]. We observed an increase in the relative abundance of *Alteromonas* in the presence of *P. astreoides* and *G. ventalina* exudates over time, with *Alteromonas* comprising most of the microbial community after growing in the presence of *G. ventalina* exudates. This observation shows how quickly *Alteromonas* can respond to coral exudates and emphasizes that exudate mixtures from different coral species can modulate diverging outcomes in overall microbial community composition. HIMB11 grew in the presence of *P. astreoides* exudates and not *G. ventalina*, suggesting that *G. ventalina* exudates do not provide HIMB11 with its required metabolites or that *G. ventalina* exudates select for microbial taxa that outcompete HIMB11 for nutrients.

Taken together, this evidence demonstrates that benthic exudates help structure reef microbial communities. This hypothesis has been introduced previously (e.g., [22]), but the results from our study, in combination with previous work, demonstrate the consistent enrichment for specific groups of microorganisms regardless of study design and location. Our work also suggests that stony corals and octocorals differentially influence seawater microbial communities, but further studies are needed to assess consistency in microbial response according to benthic functional group. To achieve this consensus, more benthic organisms need to be surveyed using the framework that we have provided here. Nevertheless, this work has important implications for understanding feedbacks between changes in the abundance of benthic organisms and responses of the surrounding reef microbial communities to those changes.

### The importance of amino acids and vitamins for reef seawater microbial communities

Using targeted metabolomics, we found that benthic exudates were generally enriched in amino acids, vitamins, and nucleosides. In oligotrophic coral reef ecosystems, these labile metabolites contain important and limiting elements like nitrogen, phosphorous, and sulfur and are likely to be rapidly incorporated by marine microorganisms. A majority of these metabolites have been measured in reef seawater [16], demonstrating that exudates from benthic organisms contribute to the metabolite pool on reefs. Amino acids are readily and efficiently consumed by marine microbial communities for biosynthesis and/or for respiration [74]. In fact, microorganisms sampled from seawater close to coral surfaces exhibited significant chemotaxis towards different amino acids, especially tryptophan [72]. In addition to other groups, microbial taxa within the family Rhodobacteraceae exhibited chemotaxis towards amino acids as well as to other compounds likely to be released by corals [72]. In our exudate addition experiments, nine Rhodobacteraceae ASVs were enriched in response to exudates from both *P. astreoides* and *G. ventalina*, although the exact ASVs differed between the two incubations and more Rhodobacteraceae were enriched in the presence of *G. ventalina* exudates (5/9 ASVs). In these incubations, Rhodobacteraceae could be responding to the availability of amino acids in exudates from *G. ventalina* and *P. astreoides*, especially tryptophan, tyrosine, and phenylalanine that were present at significant concentrations in *G. ventalina* exudates. Concentrations of measured amino acids were not significantly higher in *P. astreoides* exudates, but other amino acids could have been present and not retained by our extraction resin.

Corals can synthesize amino acids, including eight essential amino acids: valine, isoleucine, leucine, tyrosine, phenylalanine, histidine, methionine, and lysine [75]. Aligning with these results, we were able to measure detectable quantities of isoleucine, leucine, tyrosine, phenylalanine, and tryptophan in benthic exudates. Valine and lysine concentrations were also detectable, but not quantifiable due to very low extraction efficiencies. Additionally, uptake of amino acids has been previously measured in corals [76, 77] and amino acids may constitute an important source of nitrogen for corals. The amino acid tyrosine was significantly enriched in exudates from half of the species investigated (*P. strigosa, G. ventalina, R. textilis*) and was significantly depleted in *P. astreoides* exudates, potentially suggesting that *P. astreoides* holobionts were specifically incorporating background tyrosine from the incubation water. Consistent with the results of this work, tyrosine can be rapidly depleted below the limit of detection in the presence of specific coral species [77].

The concentrations of two B vitamins, riboflavin (vitamin B_2_) and pantothenic acid (vitamin B_5_) were generally elevated in organism exudates. B vitamins are important cofactors for enzymes and coenzymes that are needed to sustain cellular metabolism [78]. In marine microorganisms and eukaryotic phytoplankton, auxotrophy for B vitamins is commonly observed (reviewed within [78]), likely because of the energetic cost involved in synthesizing vitamins [79]. Instead, microorganisms who are auxotrophic for specific vitamins depend on their microbial neighbors or the environment for access to the vitamins that they require [78]. Auxotrophy for the B vitamins, cobalamin, biotin, and thiamine is common in marine microbial communities [78, 80, 81]. Auxotrophy for riboflavin has not been thoroughly investigated [80], but there is genomic evidence for pantothenic acid auxotrophy in *Pelagibacter ubique* [78, 79]. Members of the alphaproteobacterial family Pelagibacteraceae (SAR11 clade), including *Pelagibacter ubique*, are typical constituents of reef microbial communities [69, 70, 82] and exudates from *P. astreoides* enriched for the growth of SAR11 in a previous study [71]. In our work, clades within Pelagibacteraceae (identified as SAR11 Clade 1b and 1a) were present in the microbial communities of both the exudate growth and specific metabolite incubations, but there was no detectable, significant enrichment of Pelagibacteraceae in response to *P. astreoides* exudates or in response to pantothenic acid. This suggests that pantothenic acid availability is not the only factor dictating the growth of Pelagibacteraceae on coral reefs. Furthermore, there was no detectable evidence that reef seawater microbial communities responded to the presence of these two vitamins. In fact, the increasing concentrations of these B vitamins in the metabolite uptake incubations in the presence of microorganisms indicates that reef seawater microorganisms can synthesize and/or release these vitamins. Additional methods (e.g., microscopy, metatranscriptomics) should be used to explore more subtle responses of specific taxa to benthic exudates. This work demonstrates that benthic organisms can be sources of vitamins to reef microbial communities and shows that reef seawater microorganisms do not depend on benthic exudates for riboflavin and pantothenic acid, perhaps because auxotrophy for these specific vitamins is not as common and/or because neighboring microorganisms release these vitamins.

### Indole compounds in benthic exudates

Indole-based compounds, including indole-3-acetic acid (IAA), were detected in benthic exudates using both targeted and untargeted metabolomics and are likely an important group of metabolites in coral reef ecosystems. IAA is a well-studied plant auxin and phytohormone (reviewed within [83]), especially in the terrestrial realm. The production of IAA by microorganisms involved in symbiotic interactions with photosynthetic cells is common [84, 85] likely because IAA can be used to boost the photosynthetic productivity of the partner. IAA production has been implicated in facilitating marine microbial symbioses [86, 87], but the extent of IAA production in marine microbial communities as well as the ability of marine microorganisms to assimilate IAA is not well understood. In the coral exudate addition incubations, *G. ventalina* exudates enriched with high IAA concentrations stimulated the growth of *Prochlorococcus*. Our study presents preliminary evidence that *Prochlorococcus* is responding to exogenous indole-3-acetic acid released from *G. ventalina*. The genomes of some *Prochlorococcus* strains (e.g., MIT9313) contain several auxin biosynthesis genes, but the potential mechanisms of IAA import into the cell are unknown, and transporters for IAA and other auxins are not well-studied within marine microorganisms. An alternative explanation for the increase in the abundance of *Prochlorococcus* could be that *Prochlorococcus* was responding to metabolites released by the increased abundance of unpigmented cells, primarily heterotrophic bacteria, that also grew in the presence of *G. ventalina* exudates. Future work is needed to understand the capacity of *Prochlorococcus* to respond to exogenously produced IAA.

To our knowledge, this is the first report of IAA production by an octocoral holobiont. Endogenous IAA production has been observed in different types of cyanobacteria and algae (reviewed within [88]), but our findings demonstrate that *G. ventalina* and/or its associated microorganisms are capable of producing and releasing IAA as an exometabolite. IAA production and excretion could be caused by bacteria associated with Symbiodinaceae to enhance the productivity of these photosynthetic dinoflagellates within coral tissue. Alternatively, specific Symbiodinaceae that tend to associate with *G. ventalina* could also produce IAA to induce cell differentiation. More generally, MetaboAnalyst detected significant enrichment of indole-based compounds in benthic exudates compared to controls using exact mass matches to putative metabolites. This evidence suggests that benthic reef holobionts are sources of indole-based compounds. Indole-based compounds can also include indole alkaloids, a class of secondary metabolites that contain a core indole group moiety and are synthesized by an array of micro- and macro-organisms, including plants and marine organisms, typically for chemical defense (reviewed by [89]). A number of isolated indole alkaloids from marine organisms, including corals and their associated microorganisms, exhibit cytotoxic, antibacterial, anti-fouling, and antineoplastic effects [89]. While additional work is necessary to determine the identities and functions of the other indole-based compounds detected here, our results add to the existing evidence that some stony and octocoral holobionts produce and release detectable quantities of indole-based compounds.

### Specific metabolites did not impact microbial communities

We observed changes in microbial abundance and composition in response to exudates from *P. astreoides* and *G. ventalina*, but did not detect these changes in response to the individual metabolites of riboflavin, pantothenic acid, or caffeine. Our metabolite incubations were conducted using three out of thousands of metabolites that can be found in reef seawater and it is possible that we did not select metabolites that would trigger a significant response in the microbial community or that there were limitations in our methodology (16 S rRNA gene amplicon sequencing) that prevented us from detecting differences. Regardless of these possibilities, we did observe a detectable enrichment of specific ASVs in the presence of exudate mixtures from *P. astreoides* and *G. ventalina* that we did not observe in the metabolite incubations, suggesting that addition of mixed exudates elicits a more distinct community response compared to the addition of the single metabolites chosen for this study.

Our metabolite incubations included caffeine, a purine alkaloid, which we have shown here to be produced by the invasive *R. textilis* alga holobiont. Caffeine production has not been investigated widely for marine organisms, but it is a common metabolite produced by land plants, generally to deter herbivores and pathogenic microbes [90] and these features could contribute to the ability of *R. textilis* to invade and flourish on Caribbean reefs. We did not detect microbial growth inhibition by caffeine in our study of planktonic microbes, but *R. textilis* does have an unusual surface biofilm [91] that may be tolerant of and/or be able to degrade specific metabolites like caffeine. Given the growing prevalence of *Ramicrusta* on diverse Caribbean reefs [43, 44] follow-up research examining the ecological significance of its metabolites on microbes and other reef organisms is needed.

## Conclusion

This study demonstrates the importance of benthic exudates for structuring microbial communities on oligotrophic reefs by focusing on the exudates released from abundant stony corals, octocorals, and an invasive alga. We found that benthic organisms are sources of labile organic matter to the surrounding reef and that some benthic organisms like *G. ventalina* can release indole-3-acetic acid, suggesting that this specific metabolite may play an important role within coral holobionts and the surrounding reef microbial community. Furthermore, we found that exudate composition was species-specific, and various microbial taxa responded differently to exudates released from *P. astreoides* and *G. ventalina*, indicating that exudates from benthic organisms select for different members of the reef microbial community. In contrast, caffeine, pantothenic acid, and riboflavin did not select for different microorganisms, suggesting that complex mixtures of exudates from benthic organisms, rather than these individual metabolites, are important drivers of microbial community composition and function. As benthic composition continues to shift on Caribbean reefs and other reefs, the composition of exudates will change, leading to alterations in the structure and function of microbial communities and community metabolism on reefs.

## Supplementary information

Supplementary Appendix

Table S3

Table S5

## Data Availability

The datasets presented in this study are accessible on online repositories. Targeted and untargeted metabolomics data are available on the MetaboLights database under accession numbers MTBLS2855 (exudates from organism incubations) and MTBLS3286 (metabolite uptake incubations). Raw fastq files for both the exudate and metabolite uptake incubation experiments are available on NCBI’s Sequence Read Archive (SRA) and linked to BioProject PRJNA739882. Cell abundances and nutrient concentrations for the exudate uptake incubations can be accessed via the Biological and Chemical Oceanography Data Management Office (BCO-DMO) under dataset accessions 865739 and 865193, respectively. Cell abundances for the metabolite uptake incubations can be accessed at BCO-DMO under dataset accession 865159.
