# Supplementary material for: Benthic exometabolites and their ecological significance on threatened Caribbean coral reefs
Source: ISME Commun. 2022 Oct 17;2:101. doi: 10.1038/s43705-022-00184-7 (PMC9723752; doi:10.1038/s43705-022-00184-7)
Supplement: Supplementary file 1 — Supplementary Appendix [file 43705_2022_184_MOESM1_ESM.docx]

Benthic exometabolites and their ecological significance on coral reefs

Laura Weber, Melissa Kido Soule, Krista Longnecker, Cynthia C. Becker, Naomi Huntley, Elizabeth B. Kujawinski, Amy Apprill

*Supplementary Information Appendix*

1. Supplementary Methods (pages 2-8)
2. Supplementary Tables (pages 9-10)
3. Supplementary Figures (pages 11-20)

**Supplementary Methods**

*Organism exudate incubations*

Before each organism exudate incubation, 58 l of surface seawater was collected ~1 mile offshore (18 17.127° N, 064 44.312° W, 31.6 m depth) using 5 and 10 l pre-cleaned and rinsed (with seawater) Niskin bottles that were deployed by hand. The non-reef surface seawater was transferred into 3 acid-cleaned and autoclaved, 20 l polycarbonate carboys and mixed. It was then filtered using peristaltic pressure to remove cells and particles > 0.2 µm (47 mm, Omnipore, EMD Millipore Corporation, Billerica, MA, USA) and the filtrate was collected to set up the incubations and for initial solid-phase-extraction (SPE). The filtering apparatus used acid-washed PharMed BPT tubing (Masterflex, Cole-Parmer, Vernon Hills, IL, USA), Fluorinated Ethylene Propylene (FEP) tubing (890 Tubing, Nalgene, Thermo Scientific, Waltham, MA, USA), and PFA in-line filter-holders (Adventec, Cole-Parmer, Vernon Hills, Illinois, USA) to prevent plastic contamination in the samples. Immediately after filtering, two to three, 2 l subsets of the filtrate per experiment were acidified with concentrated hydrochloric acid (final concentration 1% volume/volume) and subjected to solid-phase-extraction (SPE) using a negative vacuum pressure of -3.7 to -5 100xkPA in Hg, to serve as controls. Before SPE, 6 ml, 1 gm Bond Elut PPL cartridges (Agilent, Santa Clara, CA, USA) were pre-conditioned with 6 ml of 100% methanol. For the first two incubations, filtered seawater (FSW) was collected one day prior to the experiment and refrigerated overnight. This water was removed from the fridge and equilibrated to ambient reef seawater temperature (29.5 °C) on the day of the incubation. For the other incubations, seawater was collected, filtered, and used on the same day.

*Statistical analyses of metabolomics data*

Targeted metabolite concentrations were converted to picomolar (pM) concentrations. Welch’s independent t-tests were conducted independently for each metabolite and species to assess enrichment or depletion in the organism vs. control incubations (p-values < 0.05 are significantly different). ANOVA tests were used to test for differences in concentrations of metabolites between offshore and control samples across all six incubations. If data were not normally distributed after inspection of quantile-quantile plots, Wilcoxon rank sum tests were used. Separate boxplots were made in ggplot2 (1) for each species and metabolite. To summarize the targeted data, metabolite concentrations were averaged across replicates (n=6 for organism, n=3 for control treatments) and the natural log of the fold change (log_10_[foldchange]) between the organism and control treatment was determined for each species and metabolite. Prior to this analysis, values below the limit of detection were substituted with 1/5 of the limit of detection (converted to pM) for the corresponding metabolite to avoid generating infinite values during the fold change calculations.

For the untargeted metabolomics data, filtered MS1 features were analyzed separately by ionization mode. Feature intensity matrices were corrected based on the volume of seawater that passed through each PPL cartridge and log_10_-transformed. Non-metric multidimensional scaling analysis was performed on the Bray-Curtis dissimilarity calculated between feature compositions using the R package Vegan (2). A permutational PERMANOVA test was conducted using the ‘adonis’ function in Vegan to assess which factors (species, sample type, and incubation) significantly (p<0.05) contributed to the dissimilarity between feature compositions. The adonis test was performed again using the strata argument to constrain permutations to the factors species and sample type as a way to control for drift across the 6 incubations that occurred across a time span of 1.5 weeks. MS1 features from both ionization modes were also investigated using the MetaboAnalyst 5.0 web browser (most recent publication 56). Missing values were replaced by 1/5 of the minimum positive value for each feature and intensities were transformed using a generalized log_10_ transformation. Fold changes and t-tests were conducted to determine which features were significantly (p<0.05, false discovery rate [FDR] corrected) enriched in organism compared to control incubations. Lastly, the t-test results for each feature were fed into the functional analysis module to identify putative mass matches (based on a mass tolerance of 1 ppm) to the non-lipids sub chemical class database (778 main metabolite chemical class sets) in RefMet (4) and enriched pathways using the mummichog algorithm (5) (*Table S5*).

Classical Molecular Networking (GNPS) yielded 7 unique matches out of 11 total matches. We were specifically interested in the hit to indole-3-acetyl glutamic acid (IAA-Glu) with the feature with a parent *m/z* of 388.135, because we detected indole-3-acetic acid in *Gorgonia ventalina* exudates and enrichment of indole-based compounds in benthic exudates using MetaboAnalyst. Unfortunately, the ppm discrepancy between IAA-Glu and our feature was too large and we could not identify an adduct that would account for the ppm difference. We also compared MS2 fragments between our feature (parent *m/z* 388.135), the MassBank record for IAA-Glu, and indole-3-acetic acid to query if our feature was structurally similar to either of these metabolites. We posited that our feature would be structurally similar if we could detect a major fragment of 130.0643 *m/z* in the MS2 spectra corresponding to the indole moiety + [H] adduct. Our feature had a major fragment at 130.0490 *m/z*, 120 ppm away from 130.0643 *m/z.* This amount of error is beyond the error threshold for the instrument, so we concluded that our feature 388.135 *m/z* is not structurally similar to IAA-Glu.

*Flow cytometry*

Seawater samples (1 ml) were fixed using paraformaldehyde (8%) to a final concentration of 1% (v/v), refrigerated for 20 minutes in the dark, and frozen in a charged dry shipper. Samples were shipped to the University of Hawai’i for analysis. Briefly, samples were thawed and stained with Hoechst 34442 (1 µg/mL, final concentration) (6–8) and analyzed using a Beckman-Coulter Altra Flow Cytometer endowed with two argon ion lasers, tuned to UV (200 mW) and 488 nm (1 W) excitation wavelengths. Side and forward scatter as well as fluorescence signals were collected using the appropriate filters designated for Hoechst-bound DNA, phycoerythrin, and chlorophyll. FlowJo software (Tree Star, Inc.) was used to bin populations and estimate the abundances of *Prochlorococcus*, *Synechococcus*, picoeukaryotes, and unpigmented cells (primarily heterotrophic bacteria and archaea).

*16S rRNA gene amplification, sequencing, and analyses for P. astreoides and G. ventalina exudate experiments*

To prepare libraries for sequencing on a benchtop iSeq 100 sequencer (Illumina, San Diego, CA, USA), DNA template was amplified using a two-step PCR amplification process. In the first PCR, primers 515F-Y (9) and 806R-B (10) containing Nextera P5/P7 adapters were used to amplify the V4 region of the 16S rRNA gene of bacteria and archaea. In this PCR, 1 µl (~ 1 ng µl^-1^) of purified DNA per sample was amplified using 34 reaction cycles with total reaction volumes of 25 µl. The cycle schedule was as follows: an initial 95 °C for 2 minutes followed by 34 cycles of 95 °C for 20 seconds, 55 °C for 20 seconds, and 72 °C for 5 minutes, with a final hold at 72 °C for 10 minutes. In the second PCR, Nextera XT index primers were used to attach unique indexing primers to each sample. In this step, 5 µl of purified PCR products from step one were added as template and the reaction volumes were 50 µl. The reaction schedule for the second PCR was as follows: an initial denaturation step at 95 °C for 3 minutes, followed by 8 cycles of 95 °C for 30 sec, 55 °C for 30 sec, and 72 °C for 30 sec, finished with a final extension step at 72 °C for 5 minutes. The GoTaq system (Promega, Madison, WI, USA) was used to amplify template in both PCRs and the recipe included: 1.25 units of GoTaq DNA Polymerase, 0.2 µm of the forward and reverse primers, 0.2 mm of deoxynucleoside triphosphate (dNTP) mix, 2.5 mM MgCl2, 5 or 10 μl GoTaq 5X colorless flexi buffer (depending on final reaction volume), and ultraviolet sterilized nuclease-free water. Both rounds of PCR were followed by subsequent PCR purification steps using the QIAquick 96-well vacuum manifold protocol (Qiagen). PCR products were screened using gel electrophoresis (1% agarose) after both PCRs and quantified using a Qubit 2.0 fluorometer (Life Technologies, Carlsbad, CA, USA) and the dsDNA HS assay reagents after the second PCR. Indexed and purified PCR products were diluted to 5 nM and then pooled into two different libraries to accommodate the limitations of the Illumina iSeq sequencer. The two libraries were separately diluted to 1 nM and then finally to 90 pM on the day of sequencing. A 90 pM PhiX spike-in was added to the 90 pM libraries (10% final volume) just prior to sequencing and sequencing was completed in house on the iSeq 100 following Illumina’s protocols.

*16S rRNA gene amplification and sequencing for caffeine, riboflavin and pantothenic acid* *incubation experiments*

Using 4 µl of each purified extract (due to low DNA concentrations), the V4 hypervariable region of the 16S rRNA gene of bacteria and archaea was amplified using 515F-Y (9) and 806R-B (10) barcoded primers in 50 µl reactions in preparation for an Illumina MiSeq 2x250 bp sequencing run. The PCR reaction conditions were as follows: 95 °C for 2 minutes, followed by 34 cycles of 95 °C for 20 seconds, 55 °C for 15 seconds, 72 °C for 5 minutes, and concluding with a final extension step of 72 °C for 10 minutes. The GoTaq Promega system was used to amplify the DNA using the concentrations provided above. PCR products were screened using gel electrophoresis and purified using either the QIAquick 96 PCR purification kit (using a vacuum pump) or the spin column-based QIAquick PCR purification method. PCR products were screened and quantified using the methods described above. PCR products were separately diluted to ~ 1 ng/µl concentrations in UV sterilized water and pooled to create the sequencing library. The library was sequenced using 2x250 bp Illumina MiSeq sequencing at the University of Georgia. Microbial community analysis was completed using the same methods that were documented for the exudate uptake incubations. Corncob was used to detect ASVs that responded strongly (covariance > 1) and consistently (occurring at each time point) to metabolite additions over the course of the experiment (6 and 24 hours) to discern real trends from natural variability in community composition.

*References*

1. Wickham H. ggplot2. Wiley Interdiscip Rev Comput Stat. 2011;

2. Oksanen J. Vegan: ecological diversity. R Package Version 2.4-4. 2017.

3. Chong J, Wishart DS, Xia J. Using MetaboAnalyst 4.0 for comprehensive and integrative metabolomics data analysis. Curr Protoc Bioinforma. 2019;68(1):e86.

4. Fahy E, Subramaniam S. RefMet: a reference nomenclature for metabolomics. Nat Methods [Internet]. 2020;17(12):1173–4. Available from: http://dx.doi.org/10.1038/s41592-020-01009-y

5. Li S, Park Y, Duraisingham S, Strobel FH, Khan N, Soltow QA, et al. Predicting network activity from high throughput metabolomics. PLoS Comput Biol. 2013;9(7):e1003123.

6. Campbell L, Vaulot D. Photosynthetic picoplankton community structure in the subtropical North Pacific Ocean near Hawaii (station ALOHA). Deep Res Part I. 1993;40(10):2043–60.

7. Monger BC, Landry MR. Flow cytometric analysis of marine bacteria with Hoechst 33342. Appl Environ Microbiol. 1993;59(3):905–11.

8. Campbell L, Nolla HA, Vaulot D. The importance of *Prochlorococcus* to community structure in the central North Pacific Ocean. Limnol Oceanogr. 1994;39(4):954–61.

9. Parada AE, Needham DM, Fuhrman JA. Every base matters: Assessing small subunit rRNA primers for marine microbiomes with mock communities, time series and global field samples. Environ Microbiol. 2015;18(5):1403–14.

10. Apprill A, Mcnally S, Parsons R, Weber L. Minor revision to V4 region SSU rRNA 806R gene primer greatly increases detection of SAR11 bacterioplankton. Aquat Microb Ecol. 2015;75(2):129–37.

**Supplementary Tables**

Table S1. Coral and encrusting alga collections

| Species | Collection date | Reef name | Location | Experiment date |
| --- | --- | --- | --- | --- |
| *Porites astreoides* | 11/9/2018 | Ram Head | 18.302528 N, -64.704811 W | 11/10/2018 |
| *Plexaura homomalla* | 11/12/2018 | Yawzi | 18.314360 N, -64.725910 W | 11/12/2018 |
| *Gorgonia ventalina* | 11/15/2018 | Tektite | 18.308786 N, -64.723275 W | 11/15/2018 |
| *Siderastraea siderea* | 11/15/2018 | Tektite | 18.308786 N, -64.723275 W | 11/16/2018 |
| *Pseudodiploria strigosa* | 11/16/2018 | Ram Head | 18.302528 N, -64.704811 W | 11/17/2018 |
| *Ramicrusta textilis* | 11/16/2018 | Ram Head | 18.302528 N, -64.704811 W | 11/18/2018 |

Table S2. List of deuterated metabolite standards within the injection mix

| Standard | Concentration (ng ml^-1^) |
| --- | --- |
| D2 - biotin | 50 |
| D6 – succinic acid | 150 |
| D4 – cholic acid | 50 |
| D7-indole-3-acetic acid | 150 |

Table S3. Concentrations of targeted metabolites with average concentrations and their standard deviations computed across sample groups (see separate excel file).

Table S4. Permutational adonis (permutations = 999) results conducted on untargeted data in both ion modes with constrained permutations (strata argument) and without constrained permutations.

| Ion mode | Test | Factor | Blocks (strata) | Df* | Sums Of Squares | Mean Squares | Pseudo F statistic | R^2&^ | P-value (perm.) |
| --- | --- | --- | --- | --- | --- | --- | --- | --- | --- |
| Positive | 1 | Experiment type | Incubation number | 1 | 0.0008 | 0.0008 | 3.9794 | 0.0647 | 0.001 |
| Positive | 1 | Species | Incubation number | 5 | 0.0021 | 0.0004 | 2.1104 | 0.1715 | 0.014 |
| Positive | 1 | Residuals | Incubation number | 47 | 0.0094 | 0.0002 |  | 0.7638 | - |
| Positive | 1 | Total | Incubation number | 53 | 0.0123 | 0.0021 | - | 1.0000 | - |
| Positive | 2 | Experiment type | none | 1 | 0.0008 | 0.0008 | 4.1442 | 0.0647 | 0.001 |
| Positive | 2 | Species | none | 5 | 0.0021 | 0.0004 | 2.1978 | 0.1715 | 0.001 |
| Positive | 2 | Incubation number | none | 5 | 0.0013 | 0.0003 | 1.3893 | 0.1084 | 0.005 |
| Positive | 2 | Residuals | none | 42 | 0.0081 | 0.0002 | - | 0.6554 | - |
| Positive | 2 | Total | none | 53 | 0.0123 | - | - | 1.0000 | - |
| Negative | 3 | Experiment type | Incubation number | 1 | 0.0010 | 0.0010 | 3.4584 | 0.0509 | 0.001 |
| Negative | 3 | Species | Incubation number | 5 | 0.0049 | 0.0010 | 3.4885 | 0.2569 | 0.001 |
| Negative | 3 | Residuals | Incubation number | 47 | 0.0132 | 0.0003 | - | 0.6922 | - |
| Negative | 3 | Total | Incubation number | 53 | 0.0191 | - | - | 1.0000 | - |
| Negative | 4 | Experiment type | none | 1 | 0.0010 | 0.0010 | 3.7221 | 0.0509 | 0.001 |
| Negative | 4 | Species | none | 5 | 0.0049 | 0.0010 | 3.7545 | 0.2569 | 0.001 |
| Negative | 4 | Incubation number | none | 5 | 0.0022 | 0.0004 | 1.7167 | 0.1175 | 0.001 |
| Negative | 4 | Residuals | none | 42 | 0.0110 | 0.0003 | - | 0.5747 | - |
| Negative | 4 | Total | none | 53 | 0.0191 | - | - | 1.0000 | - |

*Df = degrees of freedom

^&^R^2^ = percentage of variation explained by each factor

perm. = 999 permutations

Table S5. Enriched metabolic pathway results using MetaboAnalyst 5.0 from untargeted features ionized in positive mode and processed using XCMS (see separate excel file).

**Supplementary Figures**


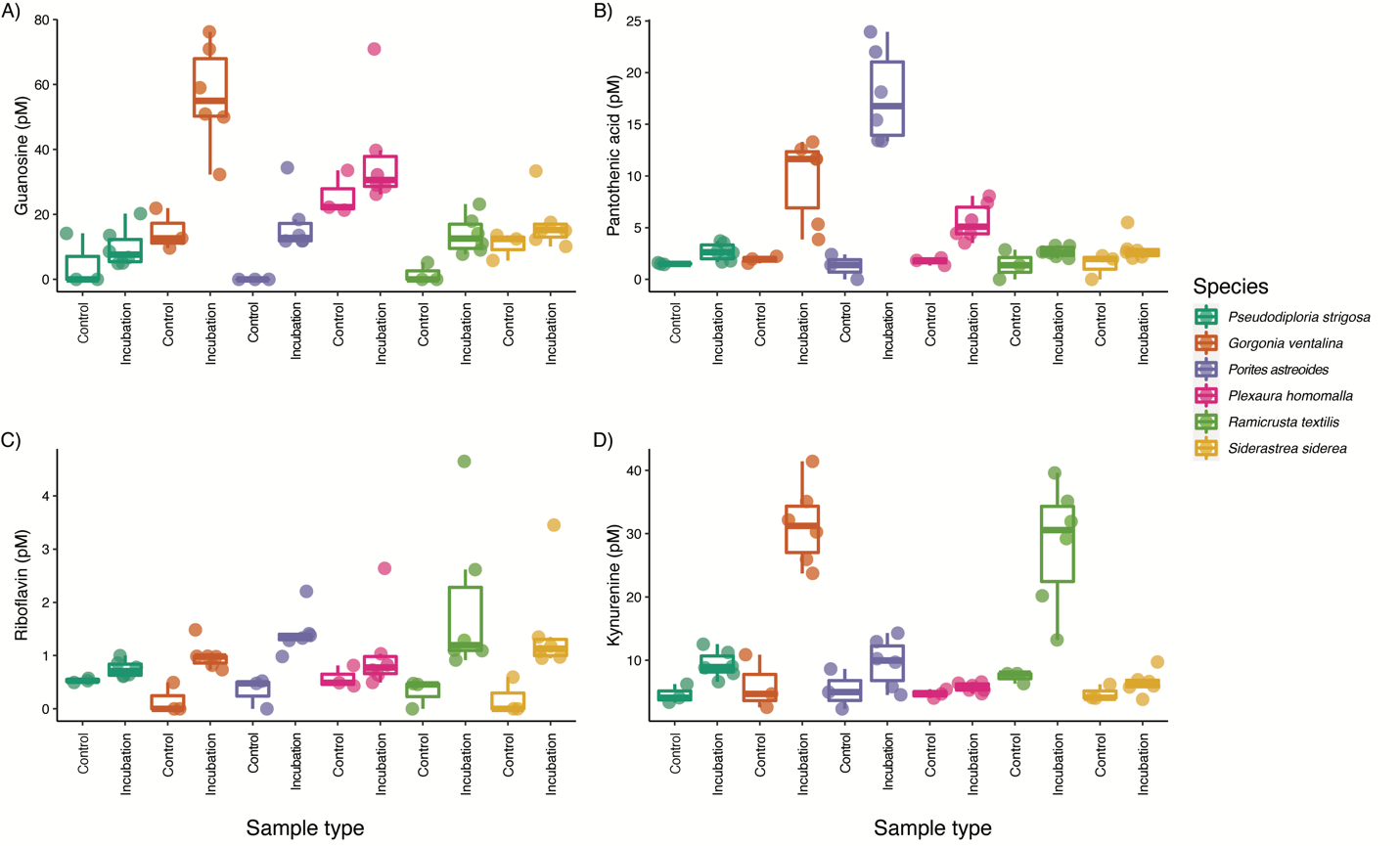


Fig. S1. Boxplots show concentrations (pM) of A) guanosine, B) pantothenic acid, C) riboflavin, and D) kynurenine across all sample types. Color corresponds to species and symbols indicate individual concentrations measured for each replicate.


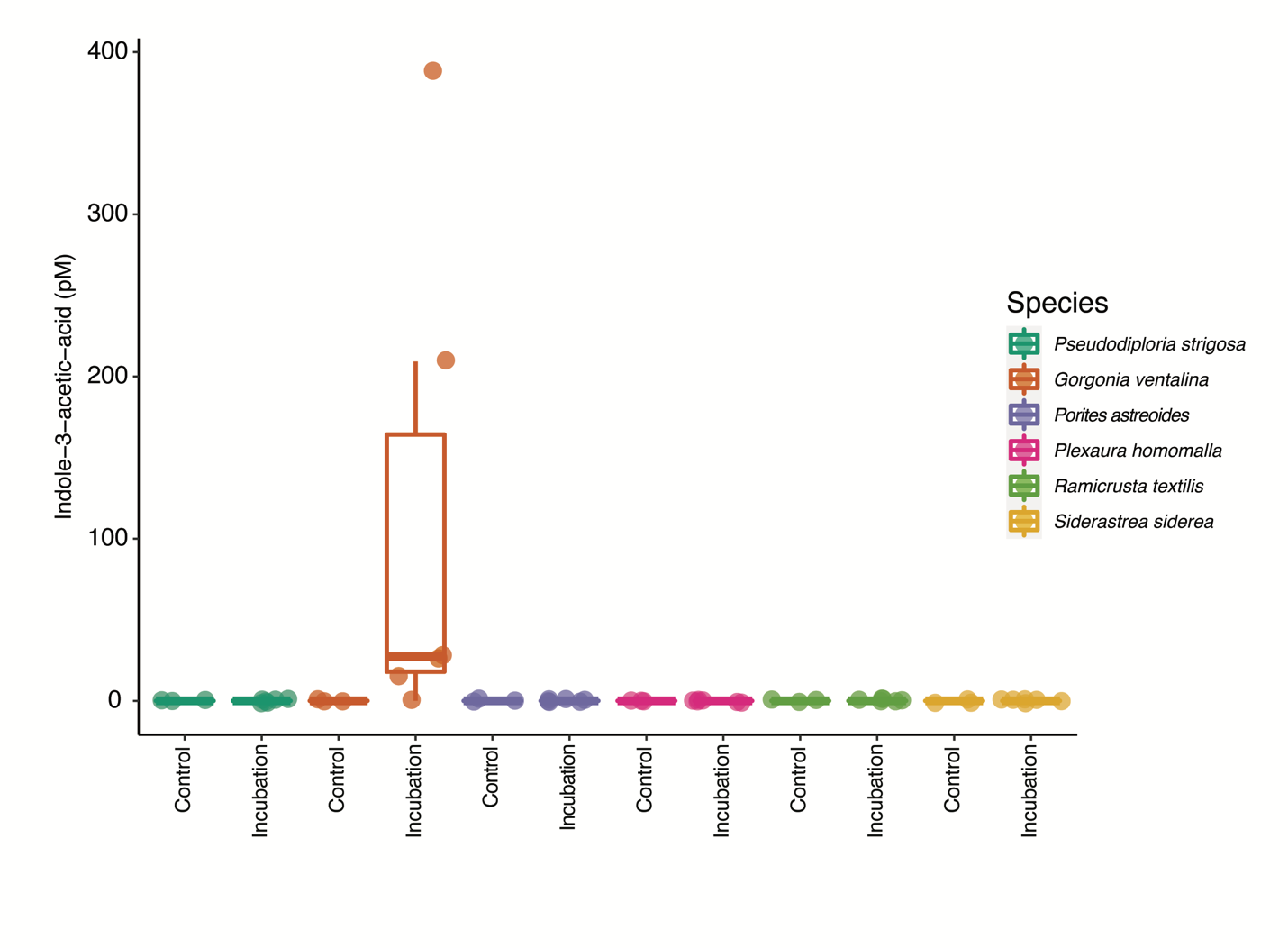


Fig. S2. Boxplots show concentrations (pM) of indole-3-acetic acid across all sample types. Color corresponds to species and symbols indicate individual concentrations measured for each replicate.


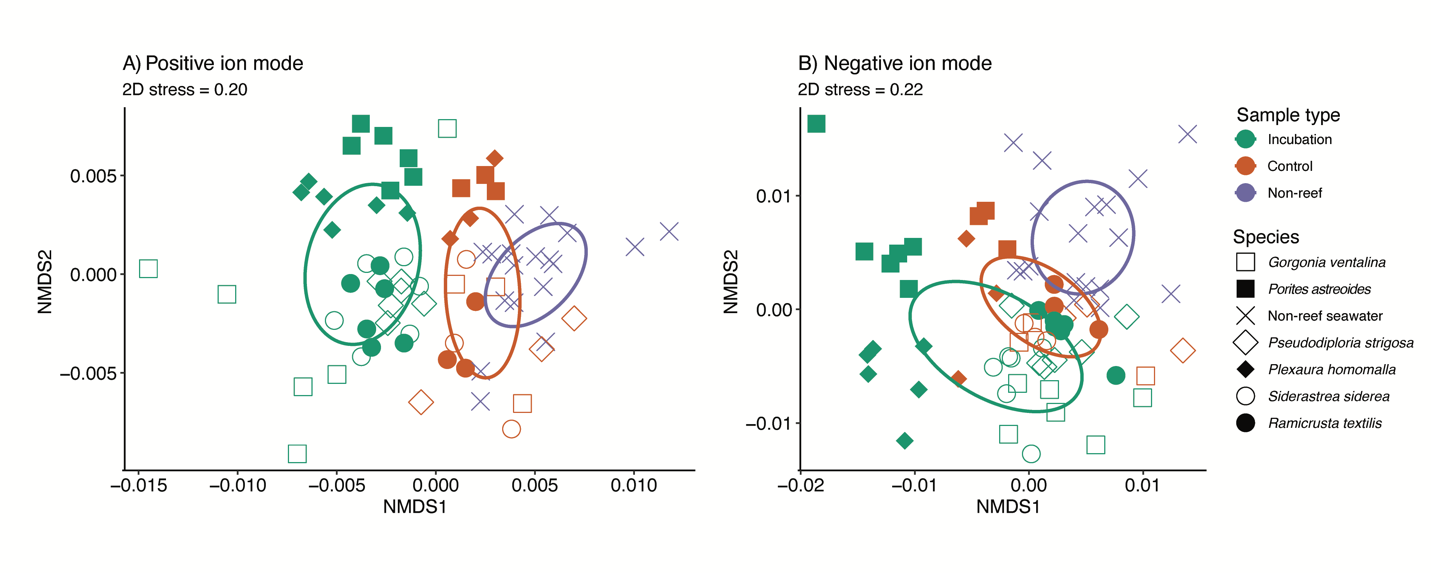


Fig. S3. Non-metric multidimensional scaling analysis (NMDS) of Bray-Curtis dissimilarities calculated for untargeted metabolite features ionized in A) positive ion mode and B) negative ion mode. This analysis includes non-reef seawater samples. Symbol shape corresponds to species and color corresponds to sample type.


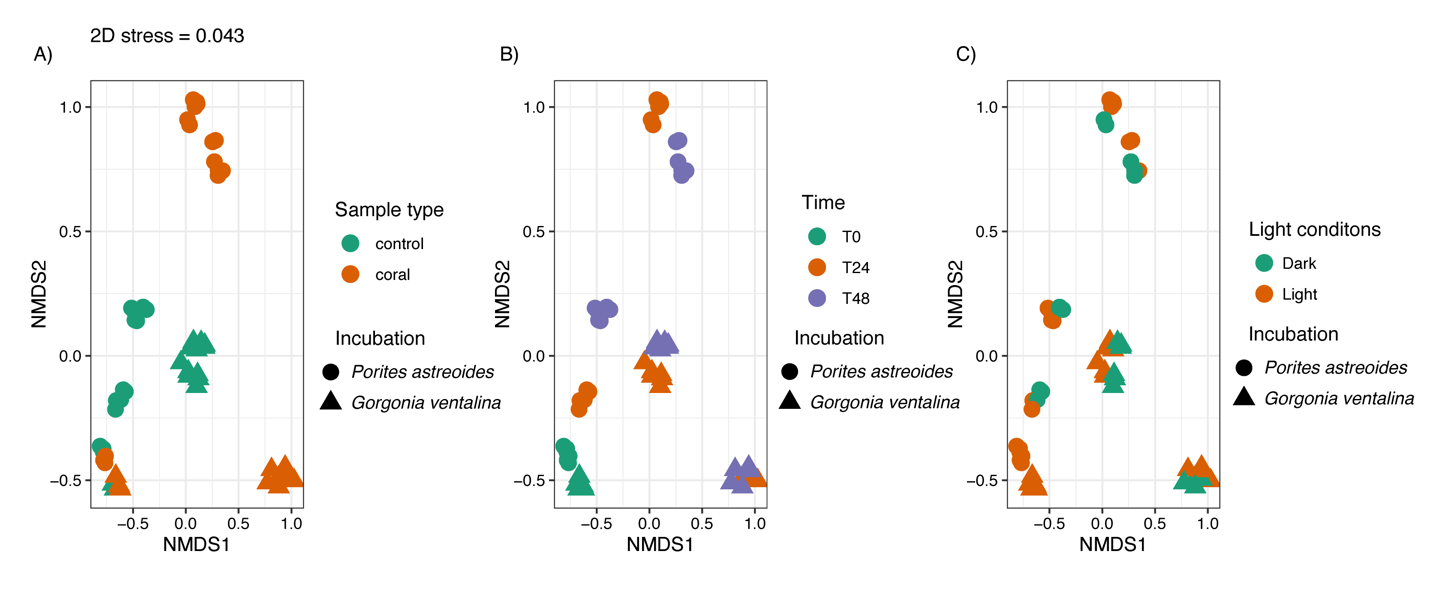


Fig. S4. Non-metric multidimensional scaling analysis (NMDS) of Bray-Curtis dissimilarities determined for microbial communities in the coral exudate incubations. In (A) symbol colors correspond to sample type (coral vs. control), (B) symbol colors correspond to the time of sample collection during the incubations, and (C) symbol colors correspond to the light conditions. In all plots, symbol shape indicates incubation type.


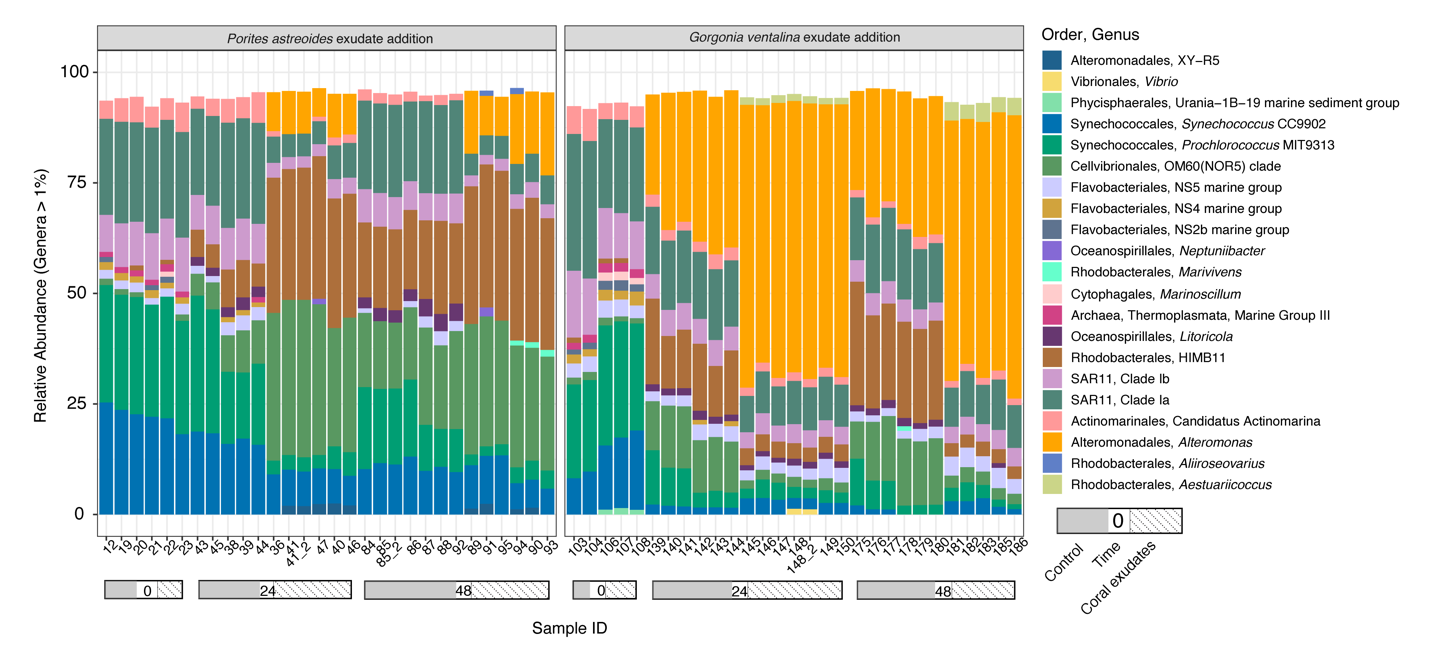


Fig. S5. Stacked bar charts displaying the relative abundances of different microbial genera in incubations across time and treatments. The bars below the x-axis indicate the time of sampling in the time series (0, 24, 48 hrs) and the treatment type: gray = control and diagonal lines = coral exudate additions. Color indicates genera.


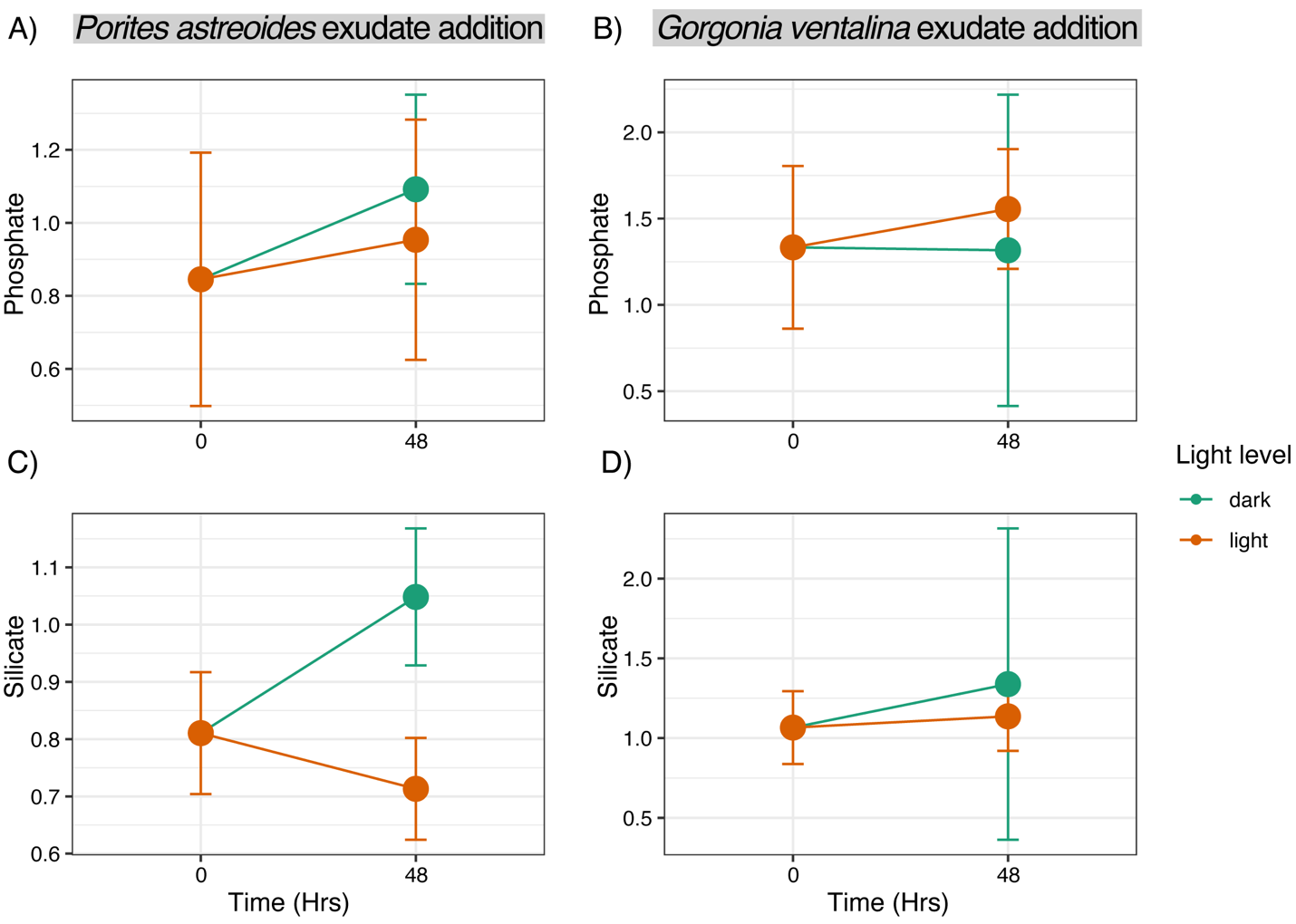


Fig. S6. Normalized concentrations of phosphate (A,B) and silicate (C,D) in the coral addition treatments for *Porites astreoides* (A,C) and *Gorgonia ventalina* (B,D) that have been normalized to the initial concentrations at 0 hrs and concentrations in the control treatments. Each circle represents the average concentration across replicates and its color indicates light level conditions.


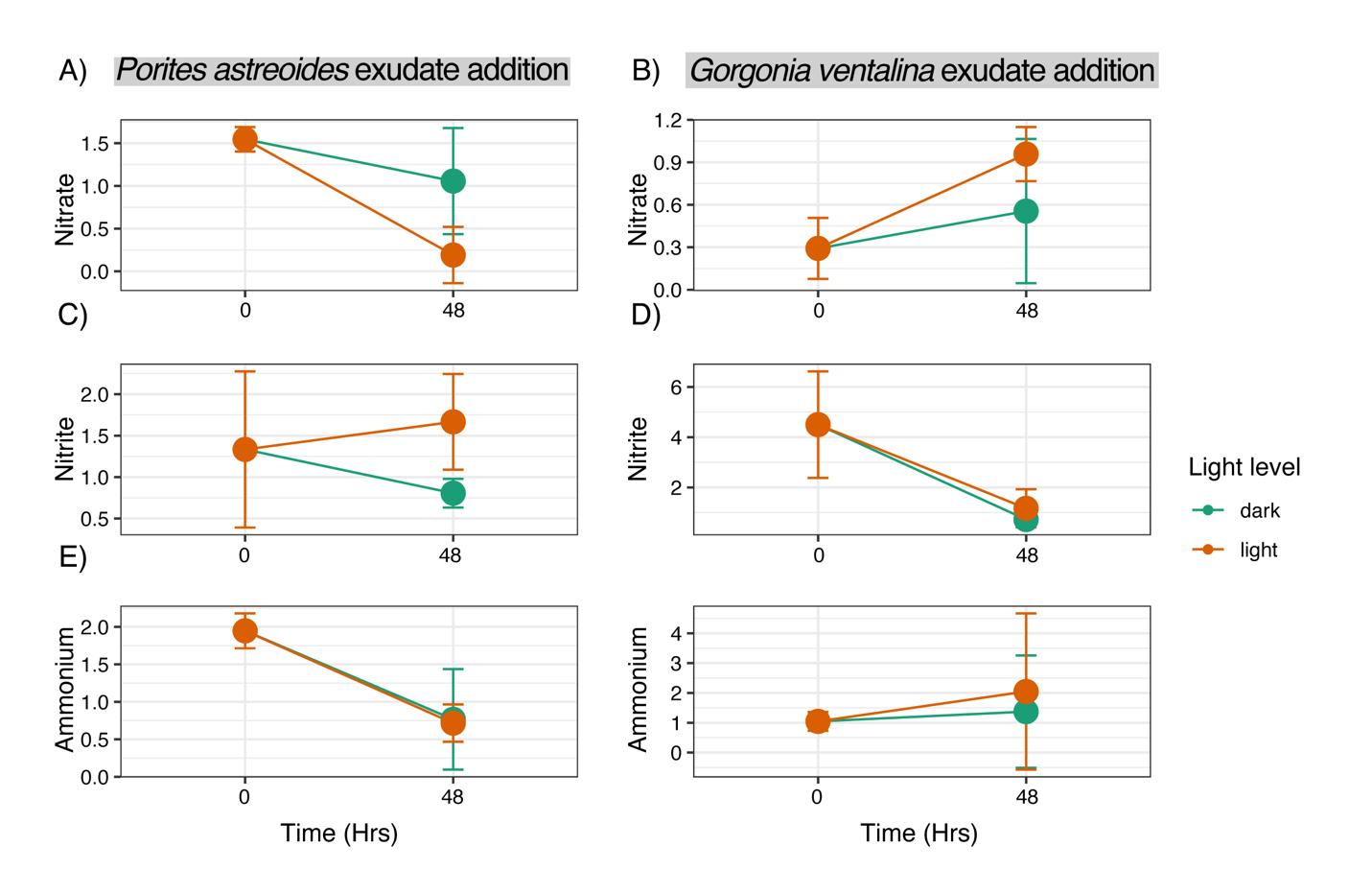


Fig. S7. Normalized concentrations of nitrate (A,B), nitrite (C,D), and ammonium (E,F) in the coral addition treatments for *Porites astreoides* (A,C, E) and *Gorgonia ventalina* (B,D, F) that have been normalized to the initial concentrations at 0 hrs and concentrations in the control treatments. Each circle represents the average concentration across replicates and its color indicates light level conditions


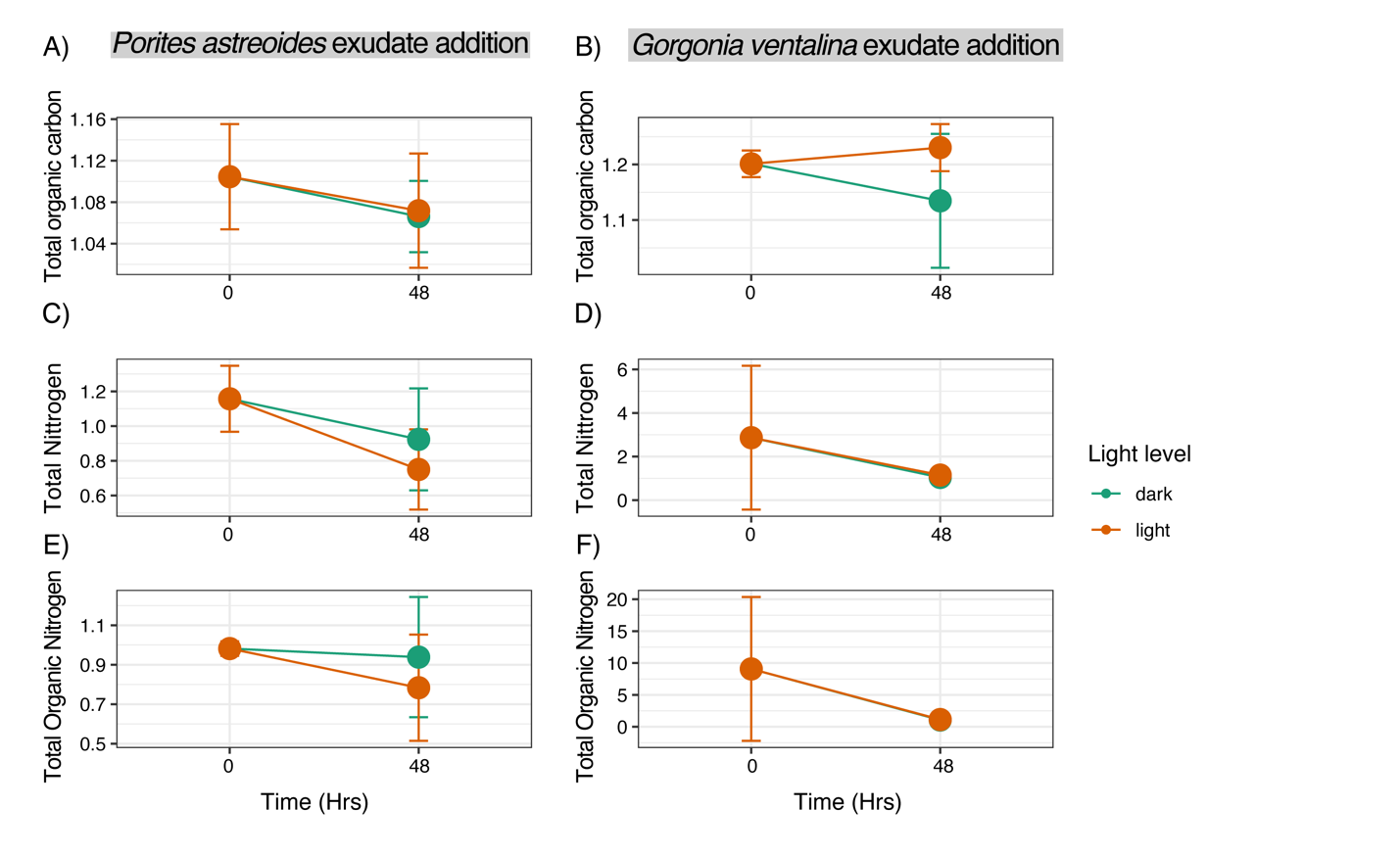


Fig. S8. Normalized concentrations of total organic carbon (A,B), total nitrogen (C,D), and total organic nitrogen (E,F) in the coral addition treatments for *Porites astreoides* (A,C,E) and *Gorgonia ventalina* (B,D,F) that have been normalized to the initial concentrations at 0 hrs and concentrations in the control treatments. Each circle represents the average concentration across replicates and its color indicates light level conditions.


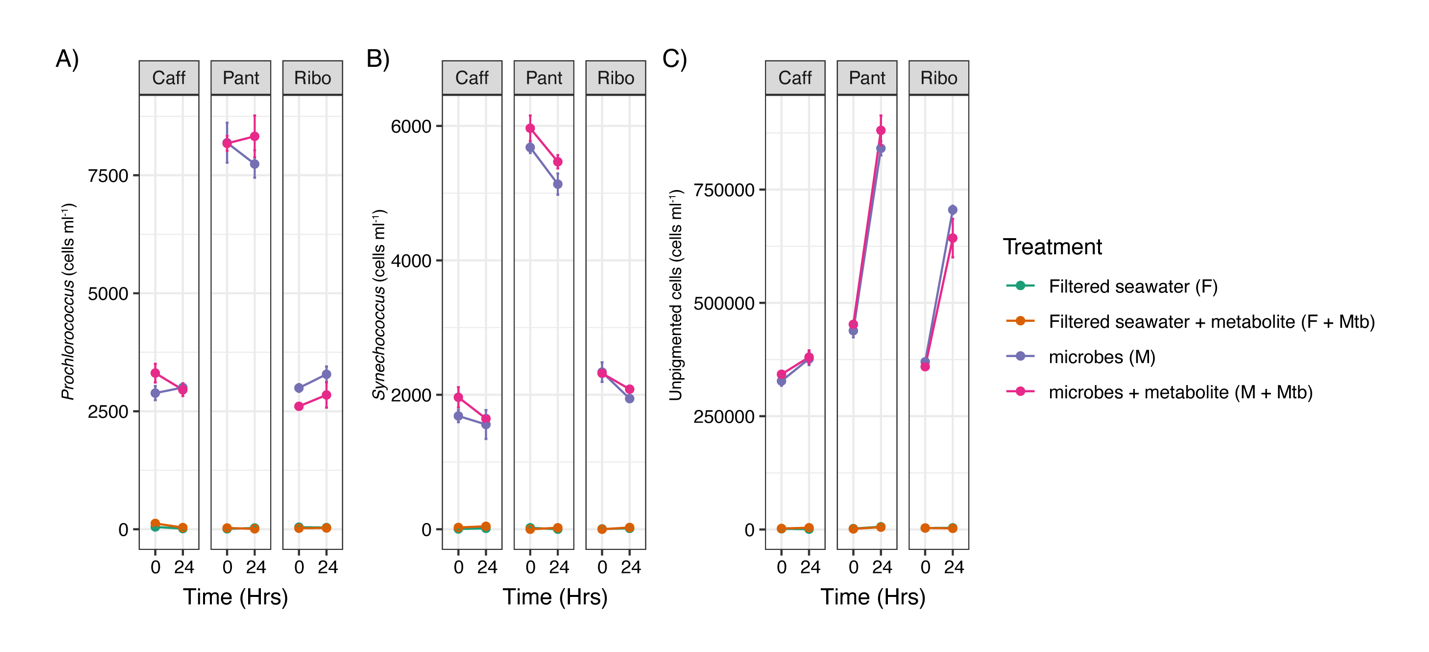


Fig. S9. Line graphs of cell counts of *Prochlorococcus* (A), *Synechococcus* (B), and unpigmented cells (C) in the presence/absence of microbes and metabolites. Color corresponds to treatment type and error bars reflect standard error across replicates for each sample type. Caff = caffeine, Pant = pantothenic acid, Ribo = riboflavin.


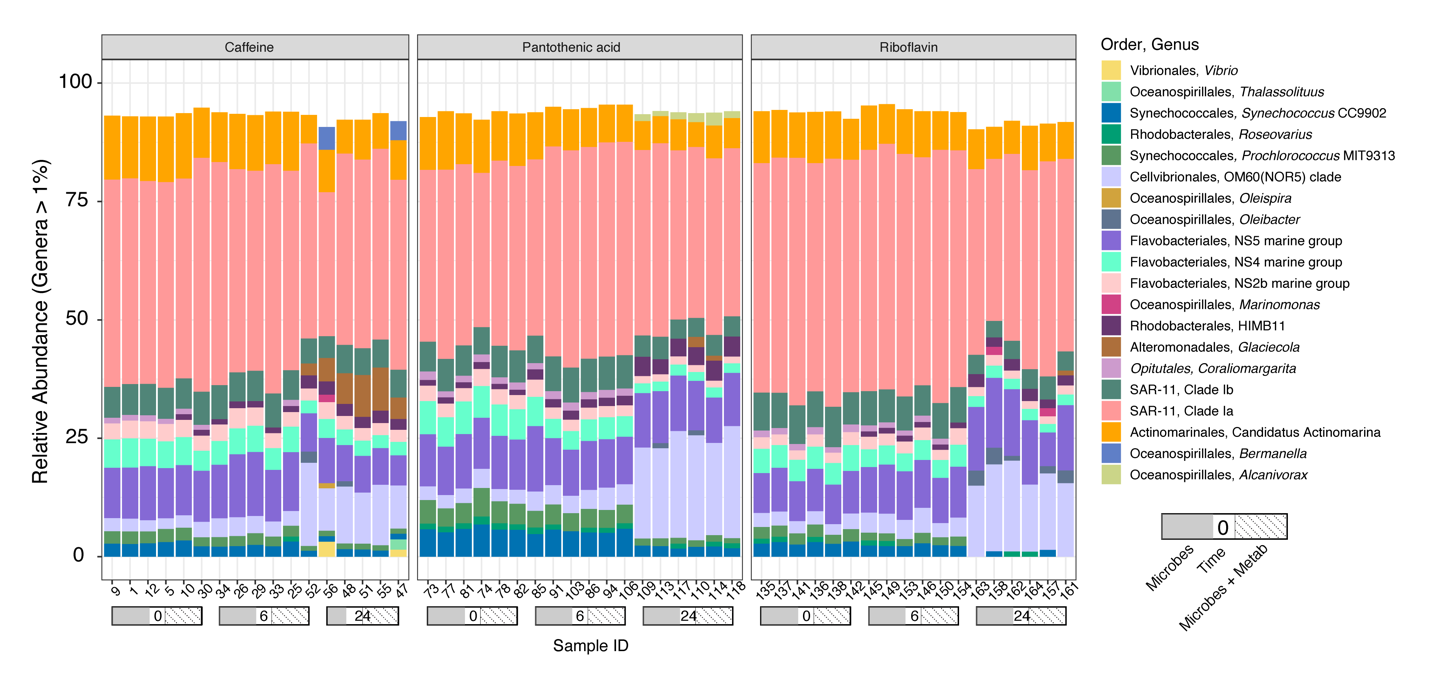


Fig. S10. Stacked bar charts displaying the relative abundances of different microbial genera in incubations across time and treatments containing microorganisms. Data is not shown for the filtered seawater treatments (F and F + Mtb). The bars below the x-axis indicate the time of sampling in the time series (0, 6, 24 hrs) and the treatment type: gray = microbes (M) and diagonal lines = microbes + metabolites (M + Mtb). Color indicates genera.
